# Supplementary material for: Stress induced TDP-43 mobility loss independent of stress granules
Source: Nat Commun. 2022 Sep 19;13:5480. doi: 10.1038/s41467-022-32939-0 (PMC9485239; doi:10.1038/s41467-022-32939-0)
Supplement: Supplementary file 1 — Supplementary Information [file 41467_2022_32939_MOESM1_ESM.docx]

*Streit et al., 2022, Supplementary Information*

*
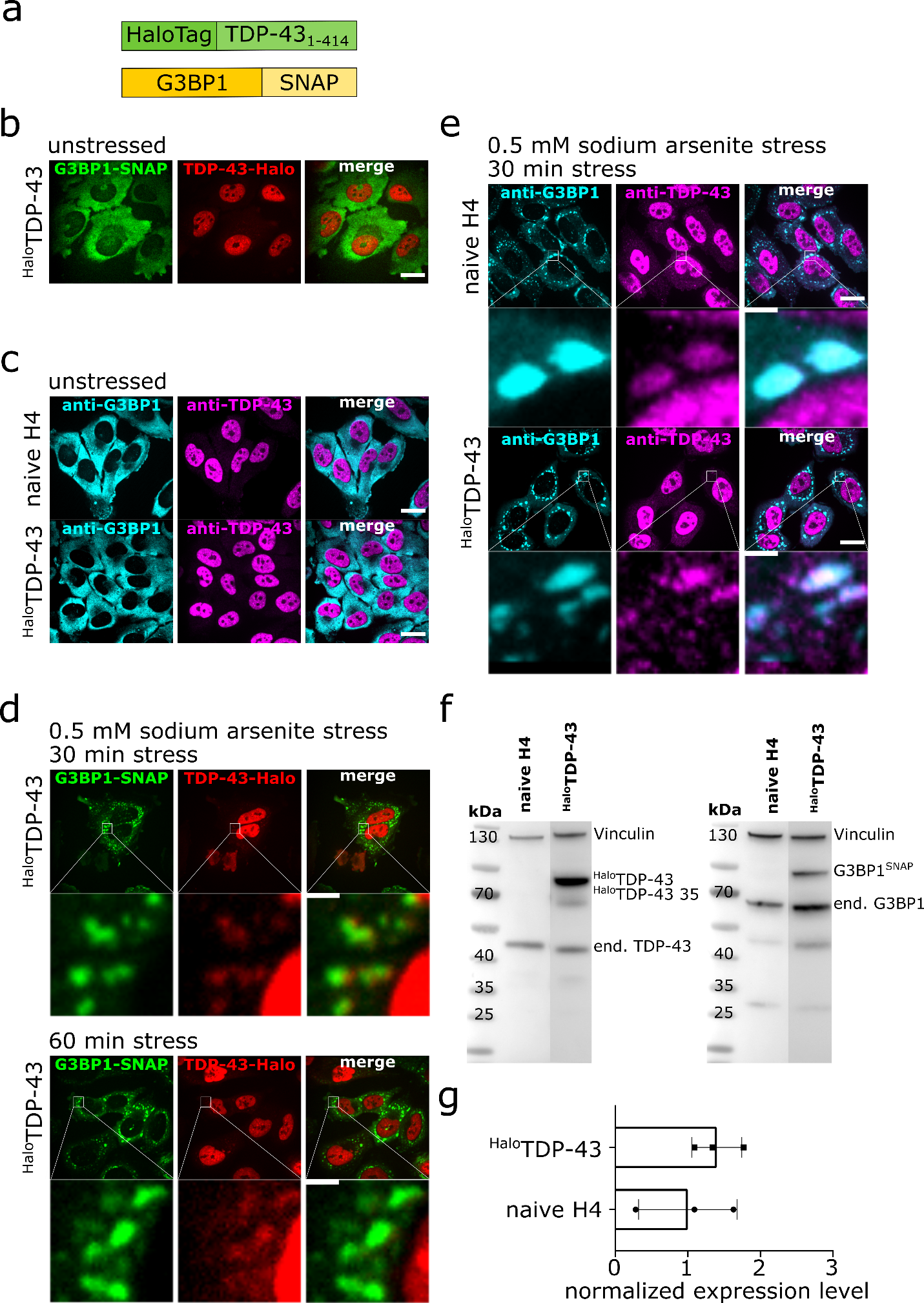
*

***Supplementary figure 1****: Generation of a ^Halo^TDP-43 wild-type cell lines.* ***a.*** *Schematic overview of the ^Halo^TDP-43 construct.* ***b.*** *Spinning disk confocal images of the ^Halo^TDP-43 cell line under unstressed conditions (red: TDP-43-TMR, green: G3BP-SiR scale bar 20 µm).* ***c.*** *Spinning disk confocal images of naïve H4 cells under unstressed conditions (cyan: anti-G3BP1-Alexa532, magenta: anti-TDP-43-Alexa647, scale bar 20 µm).* FOVs of naïve H4 cells are the same as used in figure 1 C and are only shown for clarity. ***d.*** *Spinning disk confocal images of the ^Halo^TDP-43 cell line under 30 min and 60 min sodium arsenite treatment (red: TDP-43-TMR, green: G3BP-SiR, scale bar 20 µm and 2 µm).* ***e.*** *Spinning disk confocal images of the immunostained ^Halo^TDP-43 cell line and naïve H4 cells under 60 min sodium arsenite treatment (magenta: anti-TDP-43-Alexa647, cyan: anti-G3BP-Alexa532, scale bar 20 µm and 2 µm).* FOVs of naïve H4 cells are the same as used in figure 1 E and are only shown for clarity. ***f.*** *Western Blot overview of the ^Halo^TDP-43 cell line and naïve H4 cells stained with anti-vinculin, anti-TDP-43 or anti-G3BP1 antibodies showing proper expression of the transgenic constructs.* ***g.*** *Quantification of the overexpression for the ^Halo^TDP-43 cell lines compared to endogenous TDP-43 in naïve H4 cells shows a 1.5 – 2x overexpression of ^Halo^TDP-43, n = 3 independent preparations, data are displayed as mean +/- STD. Source data are provided as a Source Data file.*


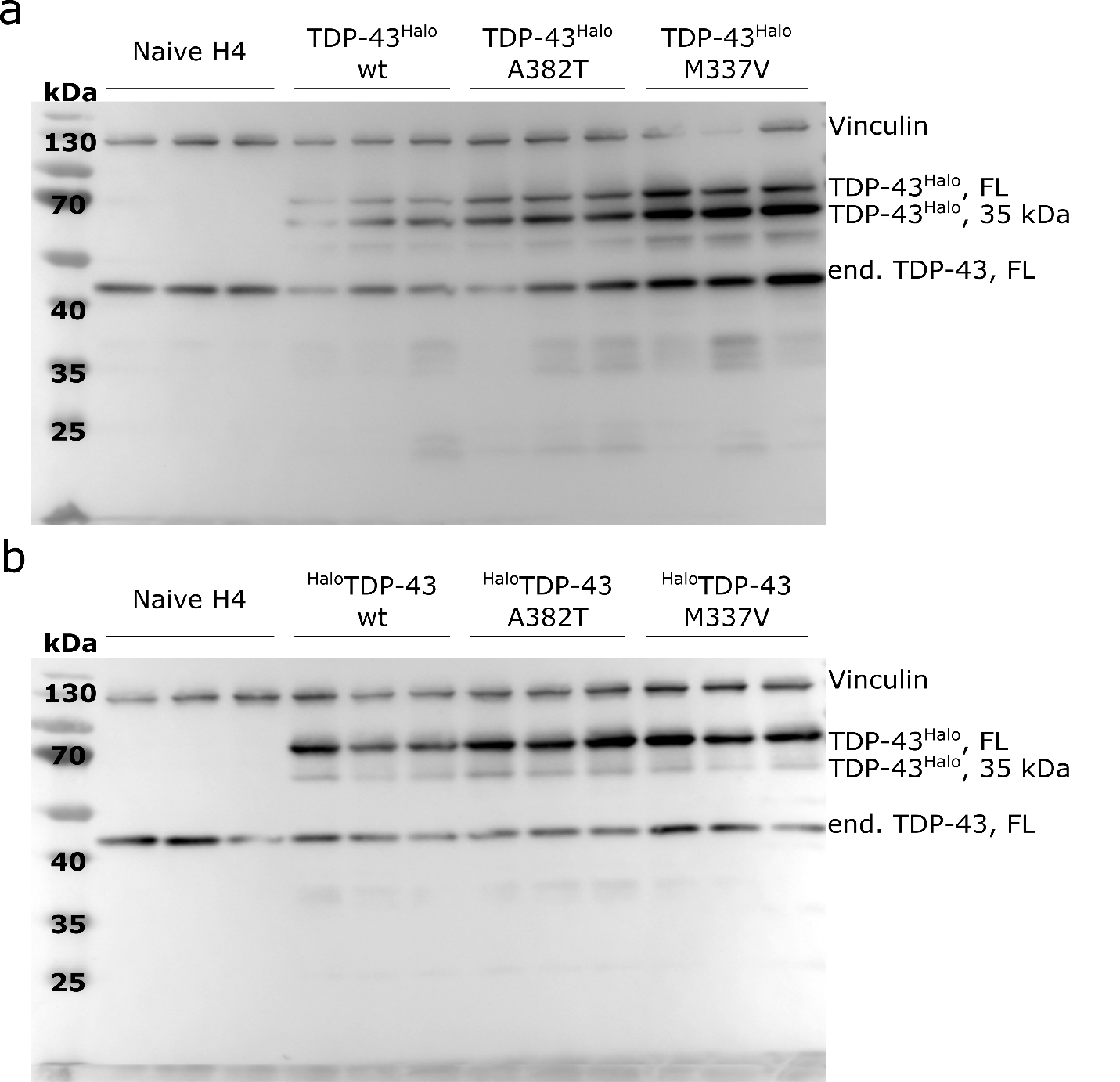



***Supplementary figure 2:*** *Western Blots for quantification of the overexpression of Halo-Tagged TDP-43 species.* ***a.*** *C-terminally tagged cell lines (wild-type, A382T, M337V).* ***b.*** *N-terminally-tagged cell lines (wild-type, A382T, M337V). Antibodies: anti-Vinculin, anti-TDP-43 (both 1:1000). The experiment was performed in triplicates.*


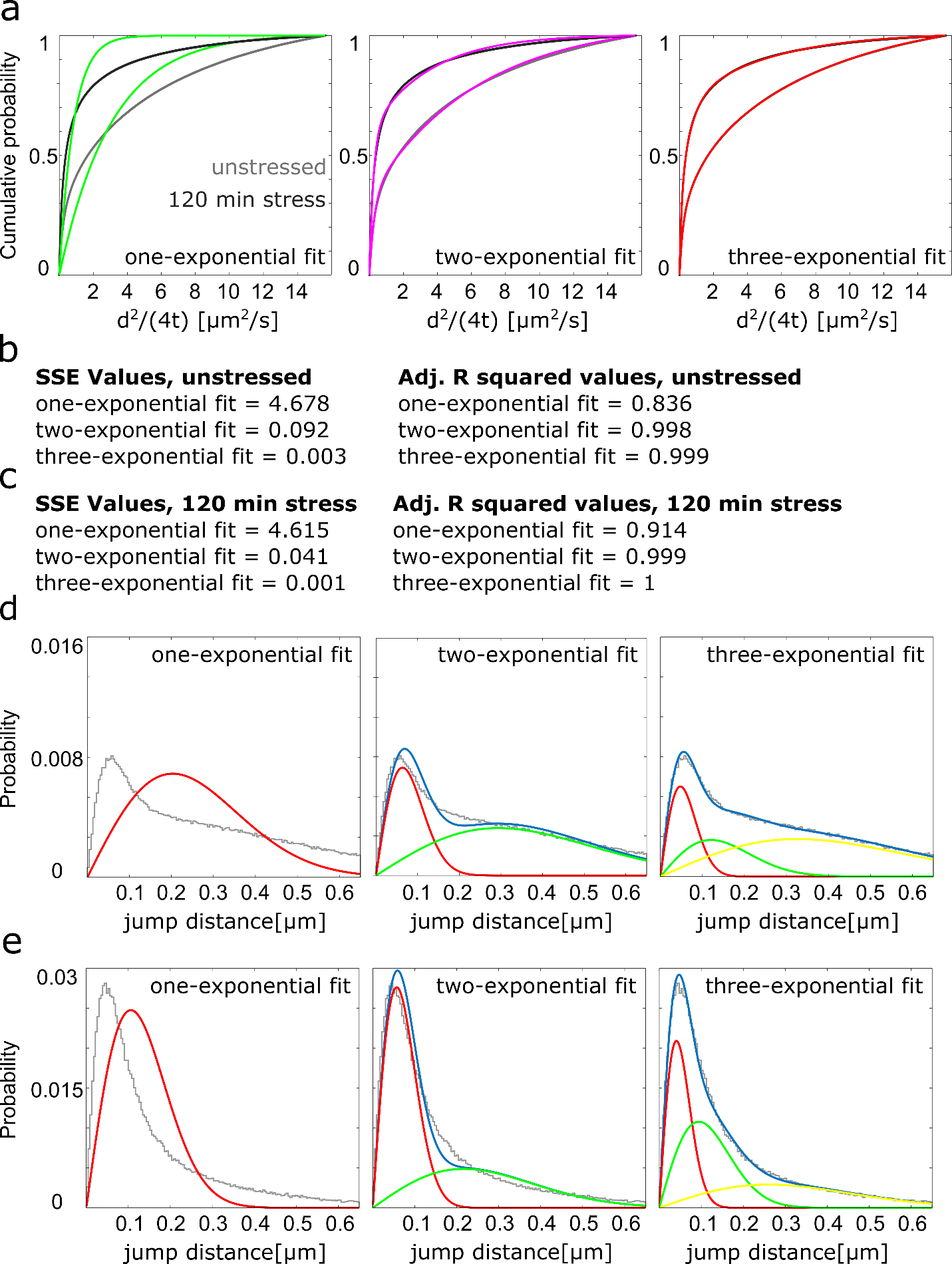


***Supplementary figure 3****: Assessment of fitting parameters for the multi-exponential fitting of cumulative distributions obtained from jump size histograms.* ***a.*** *Cumulative jump distance histogram of TDP-43^Halo^ tracks recorded under unstressed (light grey) and stressed (dark grey) conditions and fitting curves overlays (1-rate exponential fit/green, 2-rate exponential fit/magenta, 3-rate exponential fit/red).* ***b and c.*** *Overview of fit errors, SSE (summed squared error) and adjusted R^2^-error for all fitting conditions obtained after fitting of the cumulative histogram obtained from TDP-43^Halo^ tracks recorded under unstressed (light grey) and stressed (dark grey) conditions.* ***d and f.*** *Jump distance histograms obtained from TDP-43^Halo^ tracks recorded under unstressed or stress conditions overlaid with the Gaussian distributions obtained from the fitting routine.*

*
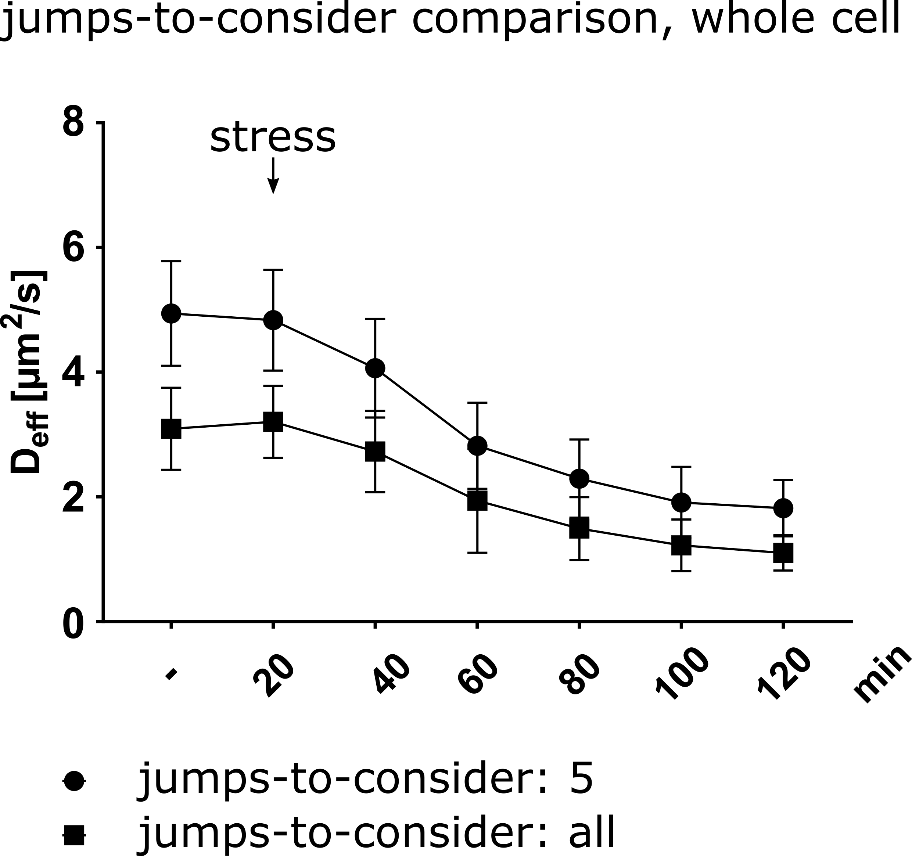
*

***Supplementary figure 4****: Comparison of the impact of the used jumps during the diffusion analysis. When using all jumps from the single-molecule tracks for each stress duration the determined D_eff_ is lower, than that determined from the first 5 jumps. This effect is well known and has been described previously* ^1,2^*. However, both procedures qualitatively show the same effect of stress-duration dependent TDP-43 slow down. The comparison was performed only for comparative reasons. The number of analyzed cells per condition (n number) is given in supplementary table S1, the experiments cells were examined in independent experiments. For all experiments, the data are presented as mean values +/- STD and the standard deviations were calculated from the movie-wise distribution of the plotted value. Source data are provided as a Source Data file.*

***
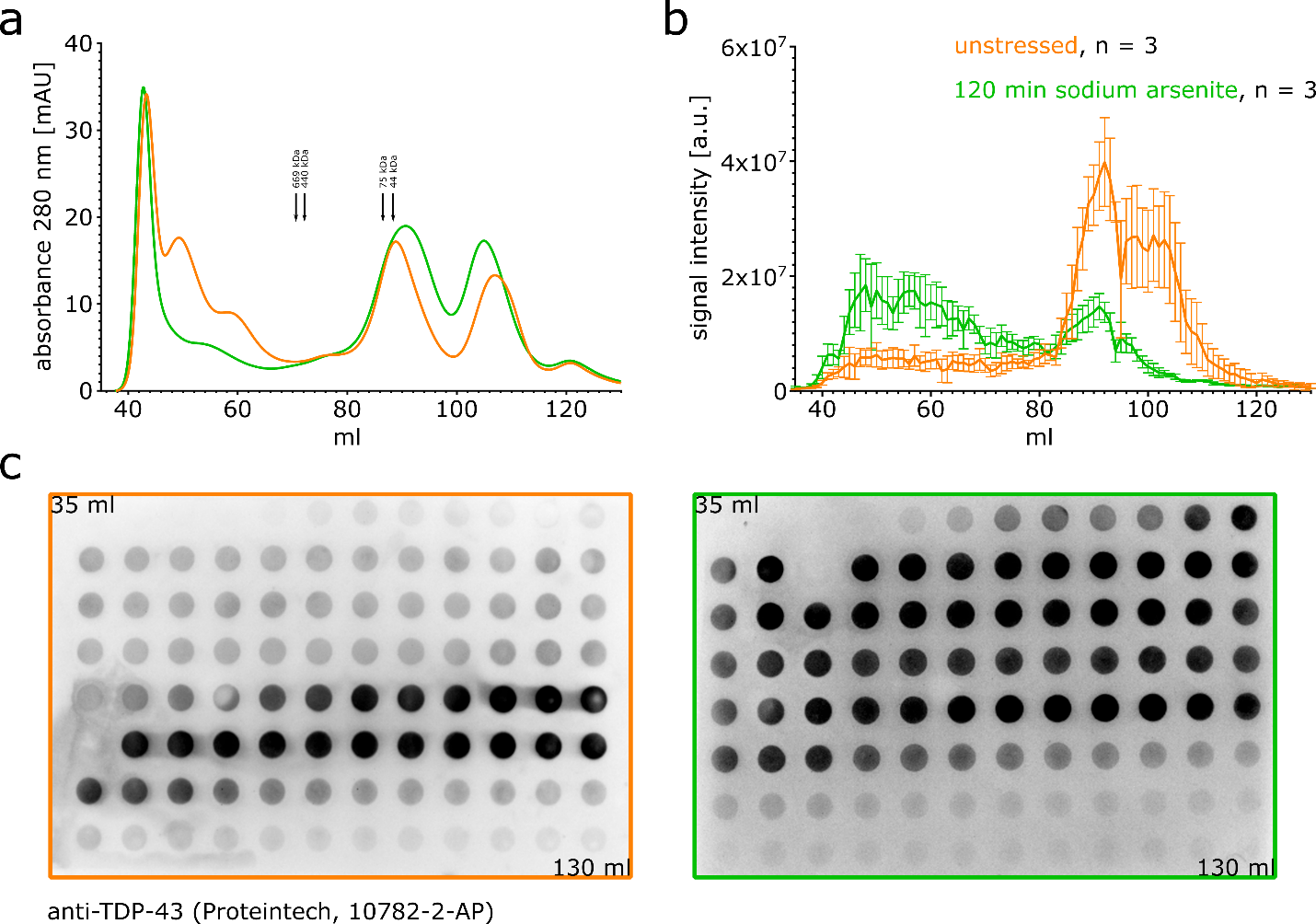
***

***Supplementary figure 5*** *Size-exclusion chromatography and dot blot for biochemically assessing TDP-43 oligomerization and aggregation under unstressed (orange) and 120 min of 0.5 mM sodium arsenite stress (green). All experiments were performed in triplicates.* ***a.*** *Chromatogram of the whole soluble protein content obtained under unstressed (orange) and 120 min of 0.5 mM sodium arsenite stress (green) conditions.* ***b.*** *Densitometric analysis of the dot blots stained for TDP-43 (antibody: anti-TDP-43, Proteintech, 10782-2-AP), Error: SEM. Source data are provided as a Source Data file.* ***c.*** *Representative dot blots for the unstressed (orange) and stressed (green) condition.*


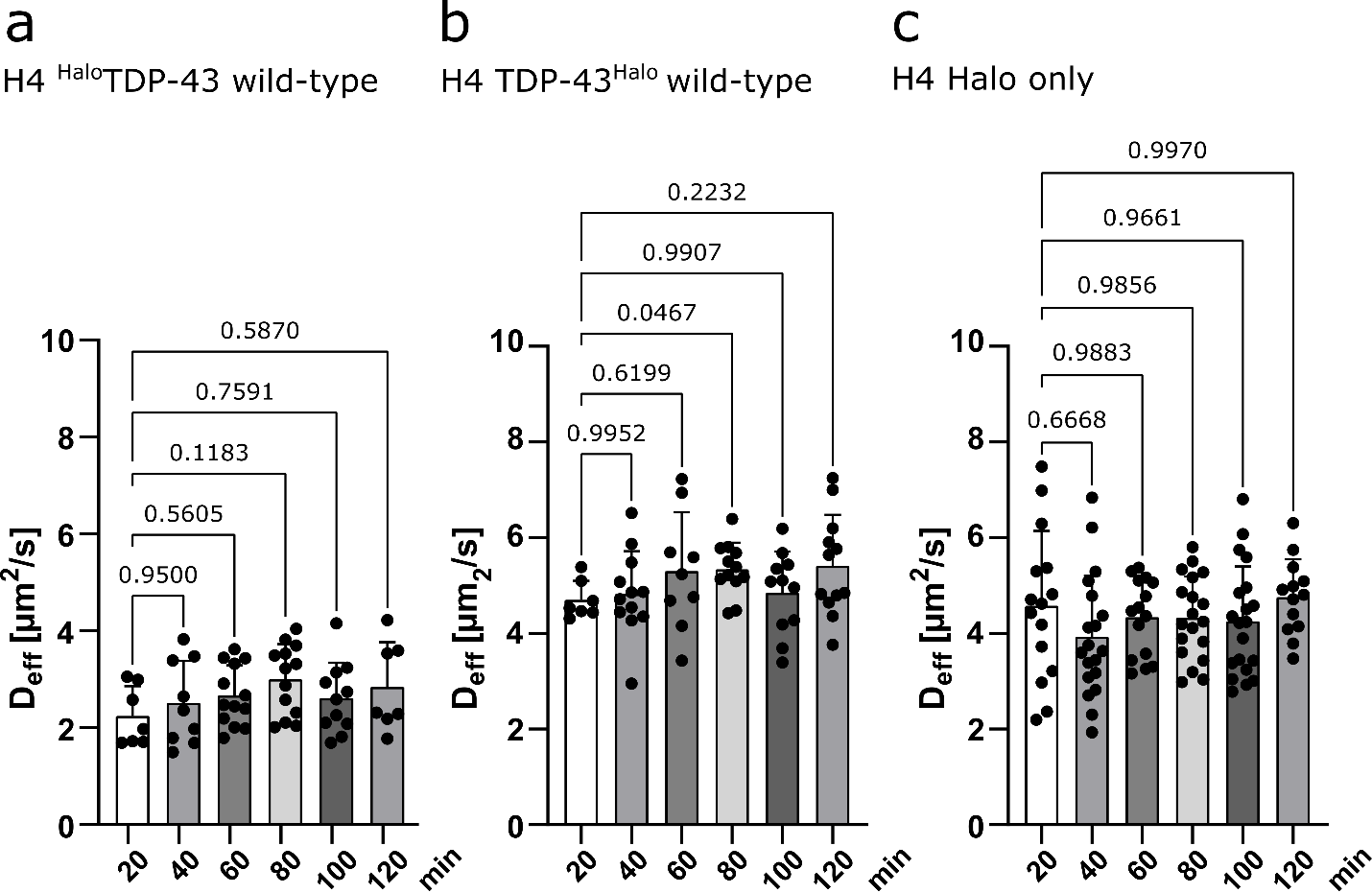


***Supplementary figure 6****: Control measurements for single-molecule tracking measurements.* ***a and b.*** *Control of C- and N-terminally tagged wild-type cell lines in absence of stress. Cells were imaged under unstressed conditions for 120 min to assess experimental procedure influences on TDP-43 mobility (Statistical test: Brown-Forsythe and Welch ANOVA, Values are displayed as the mean +/- the STD). We observed no statistically significant change in mobility during the time duration of the measurement.* ***c.*** *Halo-only stress control. Mobility was assessed for 120 min under sodium arsenite stress. The mobility of the HaloTag alone was not significantly affected when comparing different stress durations (Statistical test: Brown-Forsythe and Welch ANOVA, Values are displayed as the mean +/- the STD). Number of analyzed cells per condition (n numbers, a-c) are given in supplementary table S1. Source data are provided as a Source Data file.*


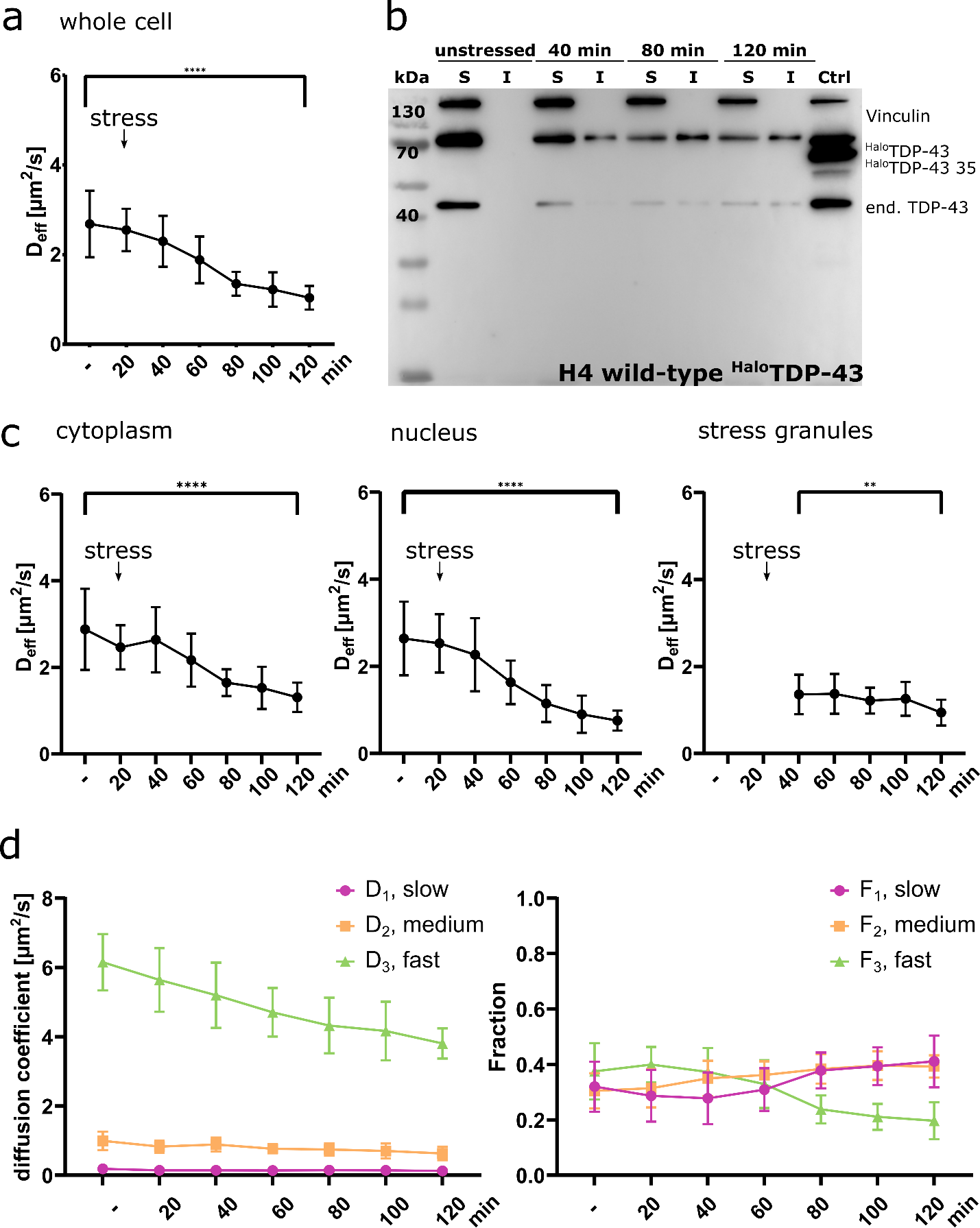


***Supplementary figure 7****: Sodium arsenite stress leads to a reduction of N-terminally tagged TDP-43 mobility in specific cellular regions (cytoplasm, nucleus, stress granules).* ***a.*** *Stress time*

*course of the effective diffusion coefficient Deff for the ^Halo^TDP-43 construct (whole cell, mean + STD, two-paired Welch’s t-test, p-value = < 0.0001). Source data are provided as a Source Data file.* ***b.*** *Solubility Assay of the ^Halo^TDP-43 construct. Solubility was assessed under unstressed conditions and different stress time-points shows an increasing insoluble TDP-43 fraction with increasing stress duration (unstressed, 40 min, 80 min and 120 min of 0.5 mM sodium arsenite treatment, anti-Vinculin, anti-TDP-43).* ***c.*** *Stress time course of the effective diffusion coefficient Deff for the ^Halo^TDP-43 construct (cytoplasm (p-value = < 0.0001), nucleus (p-value = < 0.0001), stress granule (p-value = 0.0036), mean + STD, two-tailed Welch’s t-test, n = 3). Source data are provided as a Source Data file.* ***d.*** *Analysis of the different diffusion constants (slow/D1/magenta, medium/D2/orange, fast/D3/green) and the respective fraction within the stress time-course experiment (whole cell). For all experiments, the data are presented as mean values +/- STD and the standard deviations were calculated from the movie-wise distribution of the plotted value and statistical significance was assessed with a multiple unpaired two-tailed Welch’s t-test. P-value ranges: <0.0001 ****, 0.0002 ***, 0.0021 **, 0.032 *, 0.123 ns. The number of analyzed cells per condition (n number) is given in supplementary table S1, the cells were examined in independent experiments. Source data are provided as a Source Data file.*

*
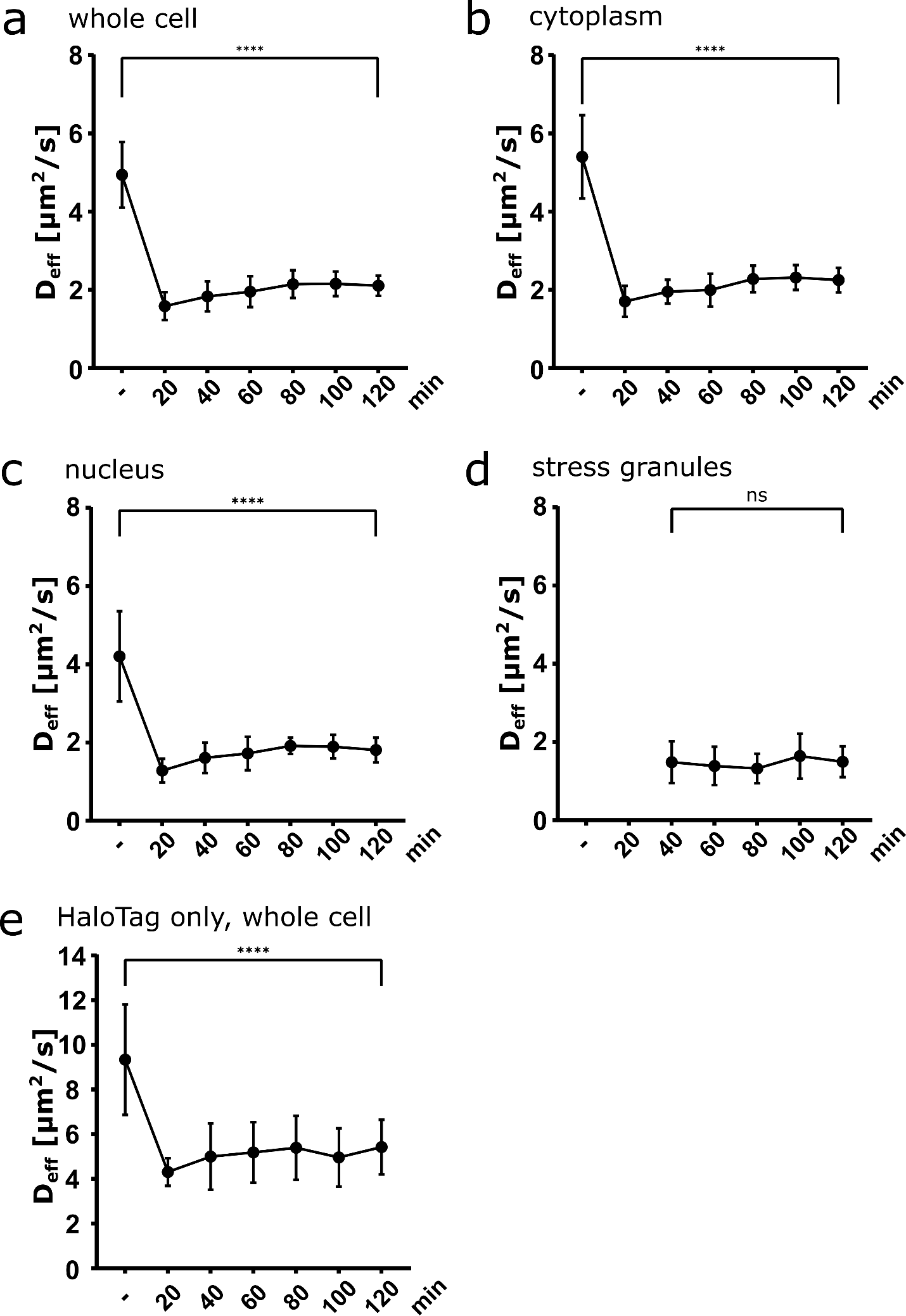
*

***Supplementary figure 8****: Sorbitol stress leads to a general reduction of TDP-43^Halo^ and HaloTag mobility in the whole cell and specific cellular regions (cytoplasm, nucleus, stress granules).* ***a - d.*** *Stress time course of the effective diffusion coefficient D_eff_ for the TDP-43^Halo^ construct (mean + STD, two-tailed Welch’s t-test). Whole cell, cytoplasm and nucleus p-value = < 0.0001, stress granules p-value = 0.9578.* ***e.*** *Stress time course of the effective diffusion coefficient D_eff_ for the HaloTag construct (mean + STD, two-tailed Welch’s t-test, p-value = < 0.0001). The number of analyzed cells per condition (n number) is given in supplementary table S1, the experiments cells were examined in independent experiments. Source data are provided as a Source Data file.*

*
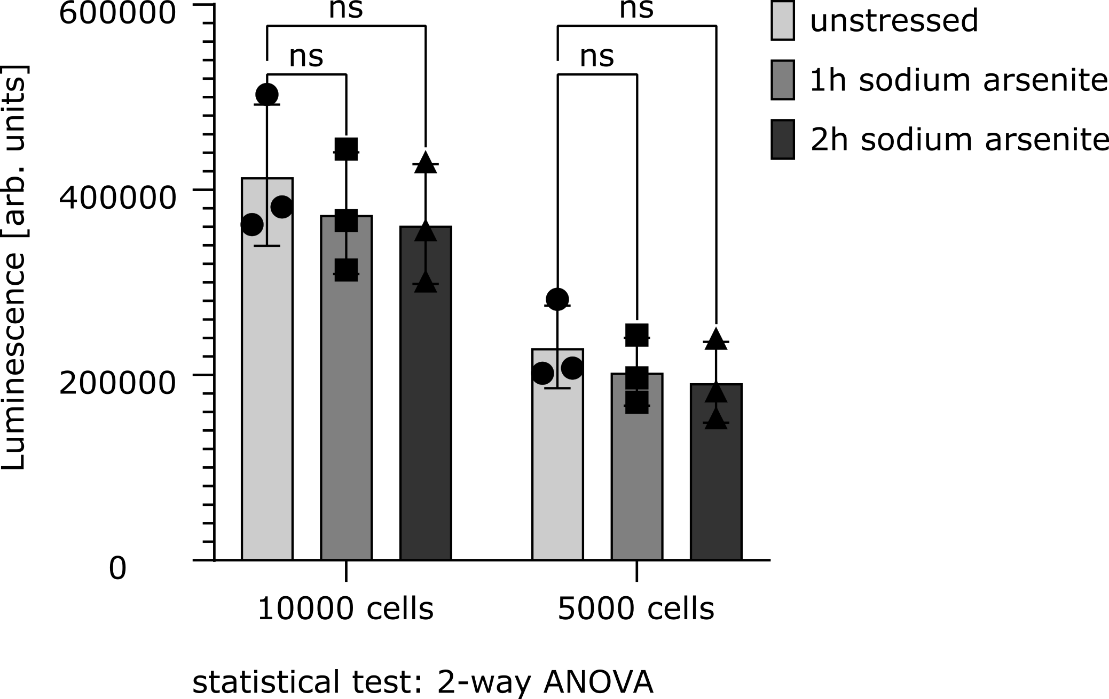
*

***Supplementary figure 9****: ATP-assay depicting cellular ATP levels with increasing stress duration. Statistical Test: 2-way ANOVA. Values are presented as mean +/- STD and were generated in n = 3 biologically independent experiments. Source data are provided as a Source Data file.*


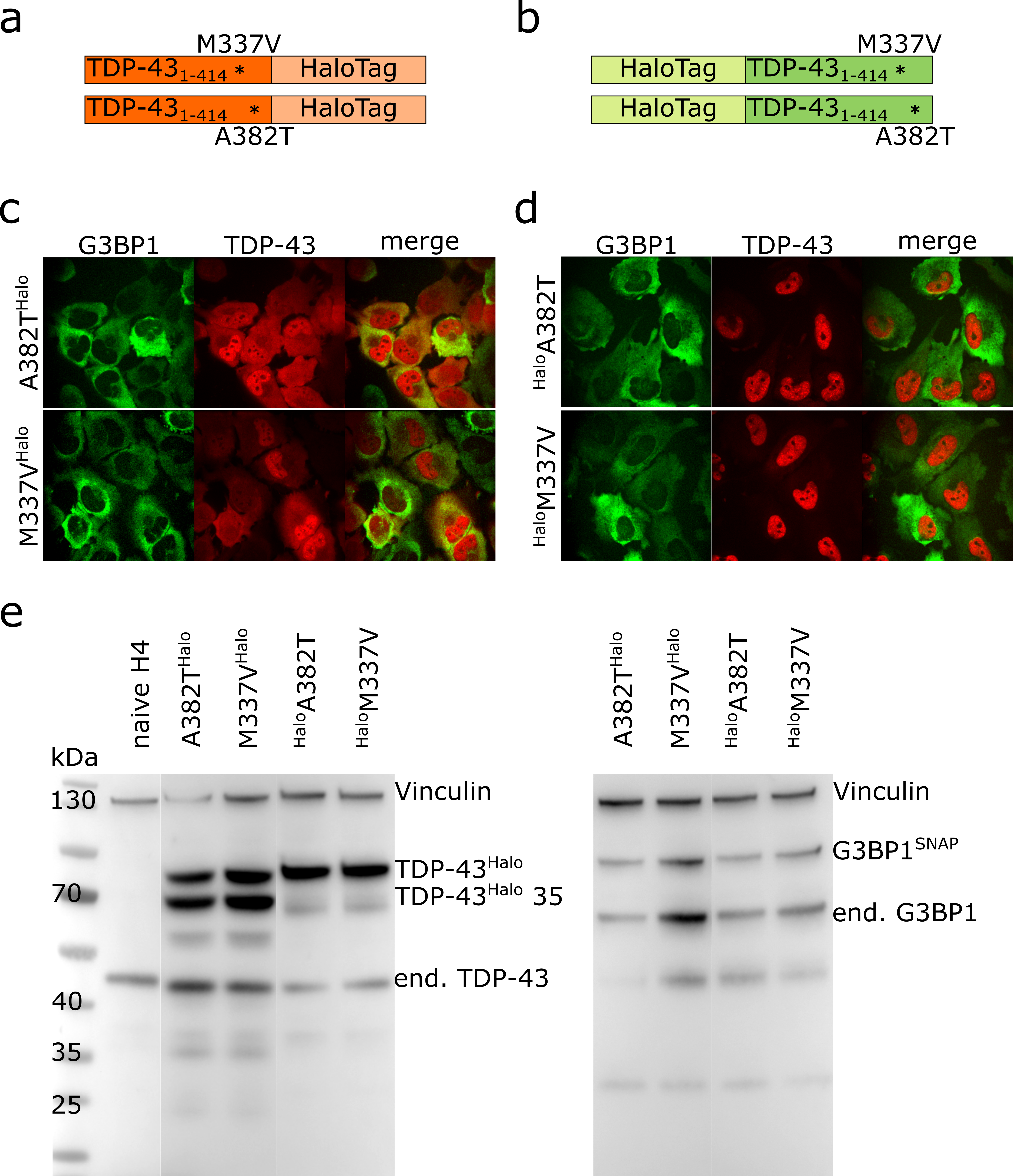


***Supplementary figure 10****: Generation of mutant TDP-43 cell lines.* ***a.*** *Schematic overview of the used TDP-43^Halo^ constructs and spinning disk confocal images of the double-transgenic (M337V^Halo^, A382T^Halo^) cell lines under unstressed conditions (red: TDP-43-TMR, green: G3BP-SiR, scale bar 10 μm).* ***b.*** *Schematic overview of the used HaloTDP-43 constructs and spinning disk confocal images of the double-transgenic cell lines (^Halo^M337V, ^Halo^A382T) under unstressed conditions (red: TDP-43-TMR, green: G3BP-SiR, scale bar 10 μm).* ***c.*** *Western Blot overview of the double-transgenic cell lines stained with anti-vinculin, anti-TDP-43 or anti-G3BP1 antibodies, n = 3.*


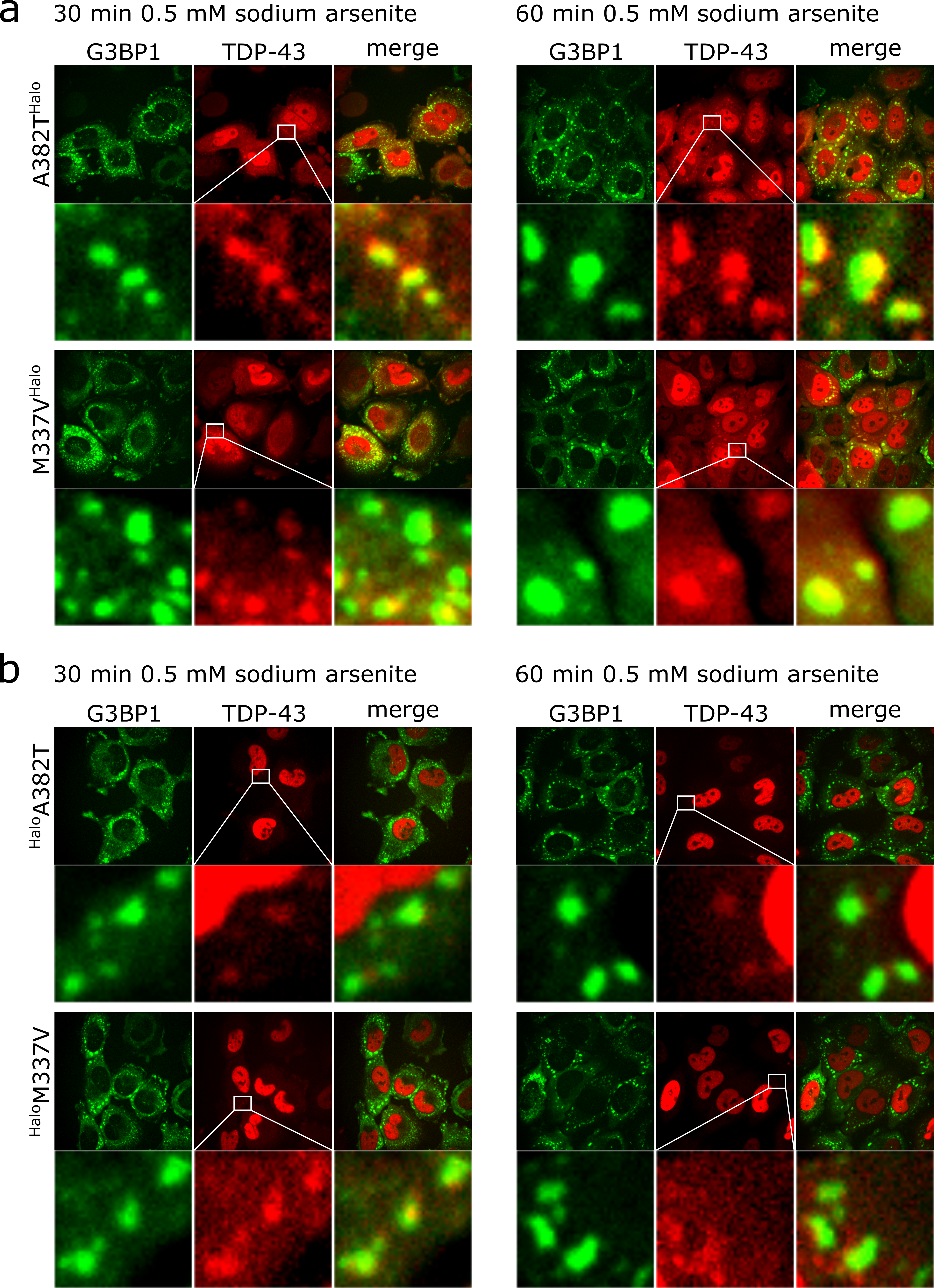


***Supplementary figure 11****: Transgenic TDP-43 constructs are found in G3BP1-positive stress granules after 30 min and 60 min of 0.5 mM sodium arsenite treatment.* ***a.*** *Spinning disk confocal images of all for all C-terminally tagged TDP-43 constructs (A382T^Halo^and M337V^Halo^ mutants) after 30 min and 60 min 0.5 mM sodium arsenite treatment. Exemplary stress granules are marked with a rectangle.* ***b.*** *Spinning disk confocal images of all for all N-terminally tagged TDP-43 constructs (^Halo^A382T and ^Halo^M337V mutants) after 30 min and 60 min 0.5 mM sodium arsenite treatment. TDP-43 granules are depicted with white rectangle (red: TDP-43-TMR, green: G3BP-SiR, scale bar 10 μm).*


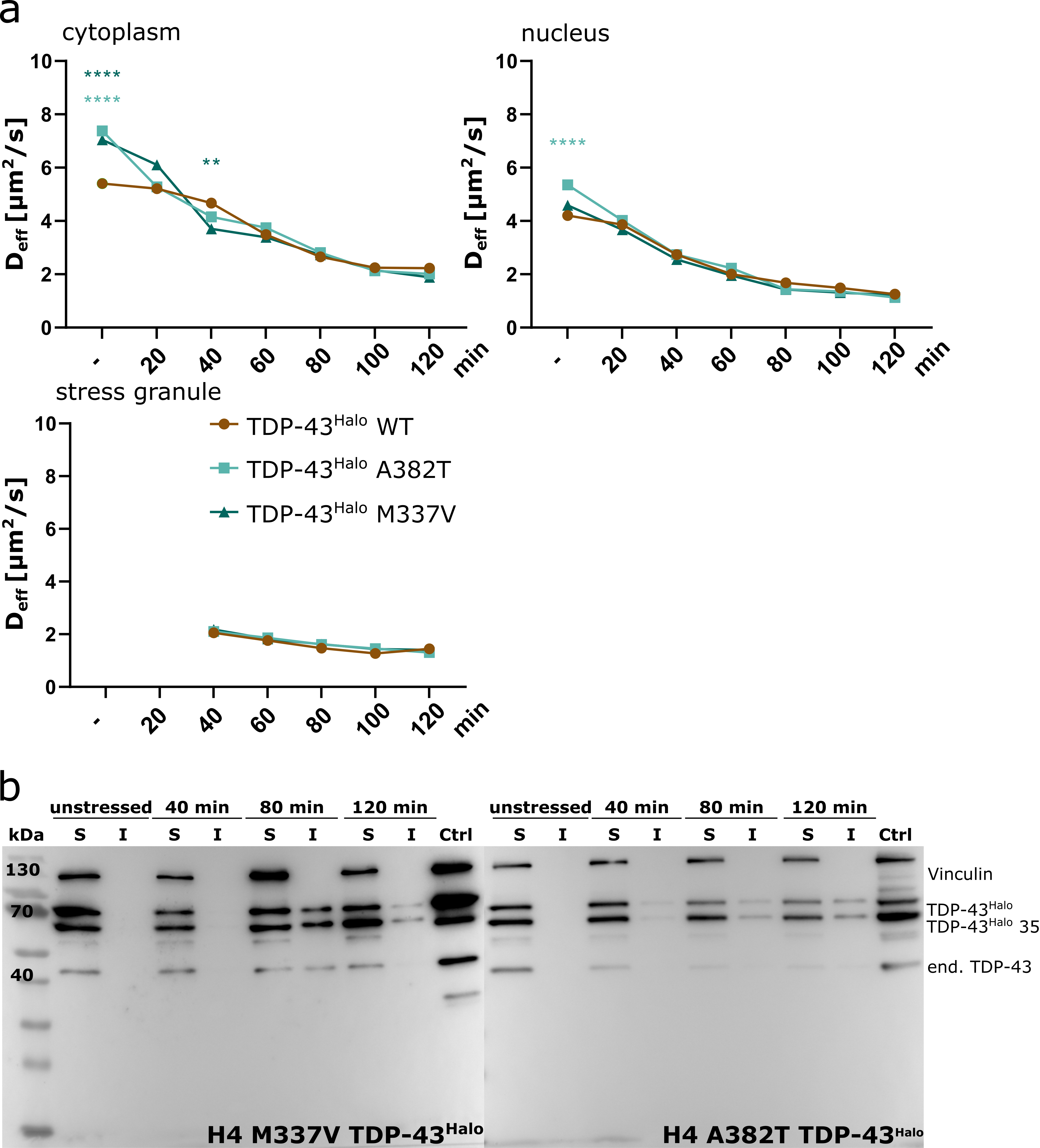


***Supplementary figure 12****: Region-wise TDP-43 mobility assessment and comparison between mutant and wild-type cell lines.* ***a.*** *Comparison of the stress related TDP-43 mobility of C-terminally tagged TDP-43 wild-type and mutant (M337V, A382T) constructs in the cytoplasm, nucleus and stress granules. Comparison shows statistical significance between the wild-type and the mutant TDP-43 at early stress conditions but no further differences at later time-points. Statistical significance was assessed by an unpaired t-test with Welch correction and was calculated between the wild type TDP-43^Halo^ construct and the respective mutant cell lines at each time point (TDP-43^Halo^ A382T, unstressed cytoplasm p-value = < 0.0001, unstressed nucleus p-value = < 0.0001, TDP-43^Halo^ M337V, unstressed cytoplasm p-value = < 0.0001, 40 min stress cytoplasm p-value = 0.0025). The number of analyzed cells per condition (n number) is given in supplementary table S1, the experiments cells were examined in independent experiments. Source data are provided as a Source Data file.* ***b.*** *Solubility assay of the C-terminally tagged TDP-43 mutants (M337V, A382T) for unstressed and stress conditions (40 min, 80 min, 120 min). Solubility assessment showed an increased insoluble fraction of both mutant TDP-43 constructs with increasing stress duration (anti-vinculin, anti-TDP-43, n = 3).*


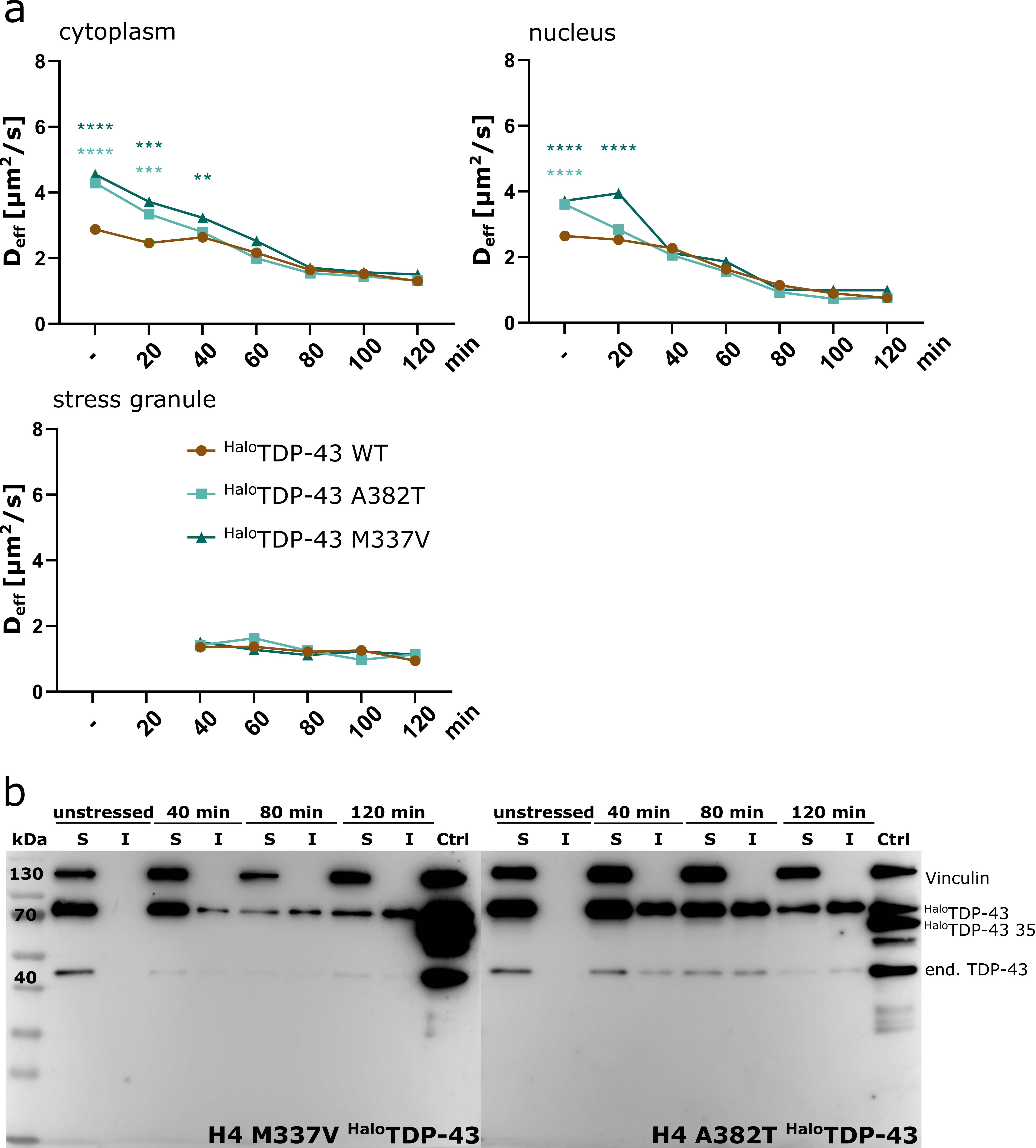


***Supplementary figure 13:*** *Region-wise TDP-43 mobility assessment and comparison between mutant and wild-type cell lines.* ***a.*** *Comparison of the stress related TDP-43 mobility of N-terminally taggedTDP-43 wild-type and mutant (M337V, A382T) constructs in the cytoplasm, nucleus and stress granules. Comparison shows statistical significance between the wild-type and the mutant TDP-43 at early stress conditions but no further differences at later time-points. Comparison shows statistical significance between the wild-type and the mutant TDP-43 at early*

*stress conditions but no further differences at later time-points. Statistical significance was assessed by an unpaired t-test with Welch correction and was calculated between the wild type TDP-43^Halo^ construct and the respective mutant cell lines at each time point (TDP-43^Halo^ A382T, unstressed cytoplasm p-value = < 0.0001, 20 min stress cytoplasm p-value = 0.0002, unstressed nucleus p-value = < 0.0001, TDP-43^Halo^ M337V, unstressed cytoplasm p-value = < 0.0001, 20 min stress cytoplasm p-value = 0.0013, 40 min stress cytoplasm p-value = 0.0057, unstressed nucleus p-value = < 0.0001, 20 min stress nucleus p-value = < 0.0001). The number of analyzed cells per condition (n number) is given in supplementary table S1, the experiments cells were examined in independent experiments. Source data are provided as a Source Data file.* ***b.*** *Solubility assay of the C-terminally tagged TDP-43 mutants (M337V, A382T) for unstressed and stress conditions (40 min, 80 min, 120 min). Solubility assessment showed an increased insoluble fraction of both mutant TDP-43 constructs with increasing stress duration (anti-vinculin, anti-TDP-43, n = 3).*


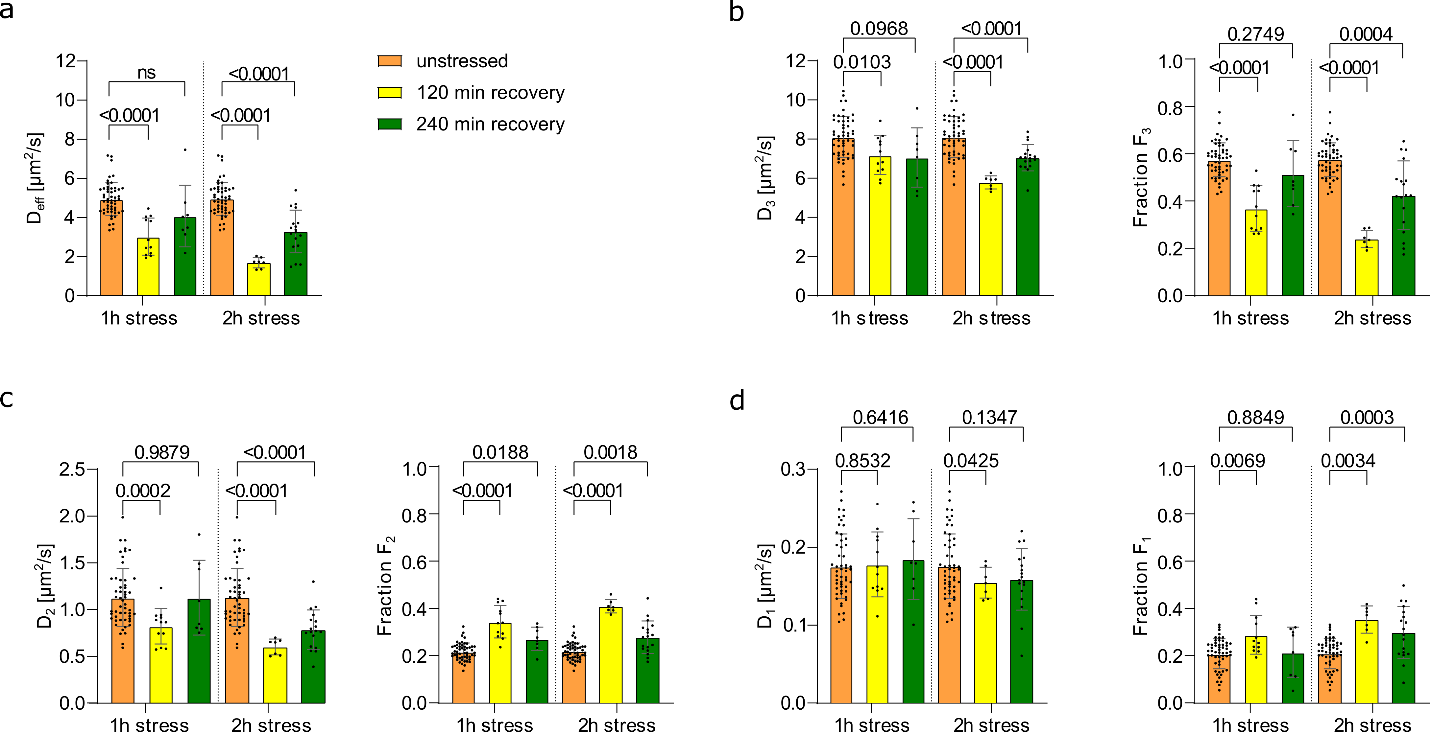


***Supplementary figure 14:*** *Statistical analysis of the effective diffusion constant Deff and the diffusion coefficients and fractions (D_3_/F_3_, D_2_/F_2_, D_1_/F_1_) of TDP-43 after 60 min or 120 min of sodium arsenite stress and 120 min or 240 min of recovery (orange: unstressed, yellow: 120 min recovery, green: 240 min recovery). Statistical significance was assessed with an unpaired two-tailed, t-test with two-tailed Welch’s correction.* ***a.*** *Comparison of Deff between unstressed conditions and 120 min or 240 min of recovery after 60 min or 120 min of stress.* ***b – d.*** *Comparison of D_3_/F_3_, D_2_/F_2_ and D_1_/F_1_ after 60 min or 120 min stress at unstressed condition, 120 min or 240 min of recovery. Statistical significance was assessed with an unpaired two-tailed, t-test with two-tailed Welch’s correction. P-value ranges: <0.0001 ****, 0.0002 ***, 0.0021 **, 0.032 *, 0.123 ns. For all presented data, the number of analyzed cells per condition (n number) is shown in supplementary figure S1. For all experiments, the data are presented as mean values +/- STD and the standard deviations were calculated from the movie-wise distribution of the plotted value. Source data are provided as a Source Data file.*


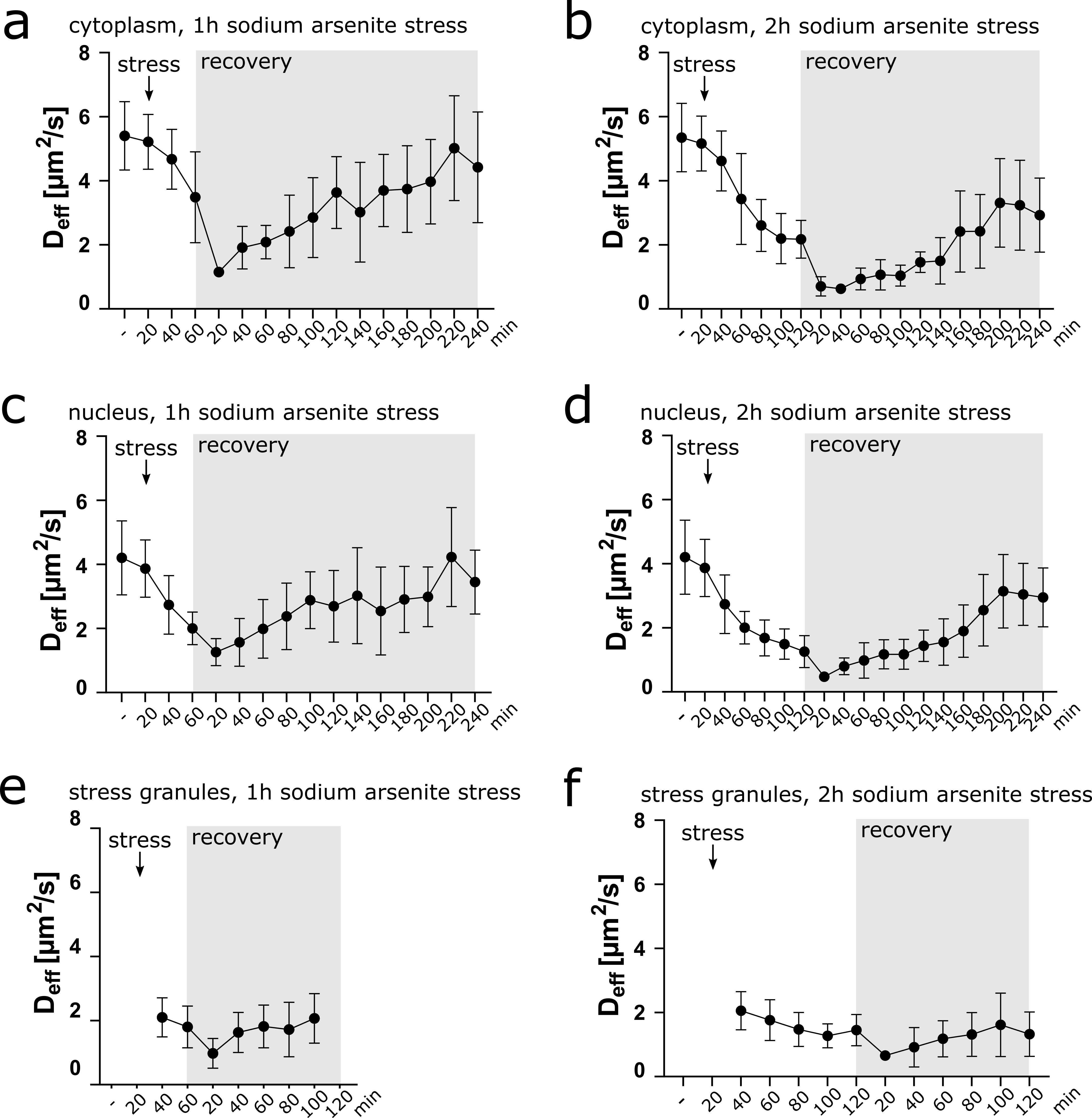


***Supplementary figure 15:*** *Region specific analysis of the stress and recovery experiments.* ***a, c and e:*** *Stress and recovery time courses of the effective diffusion coefficient D_eff_ plotted for the whole cell for 1h stress duration and up to 4h of recovery. Stress start points are marked by arrows and the recovery period is highlighted in grey.* ***b, d and f:*** *Stress and recovery time courses of the effective diffusion coefficient D_eff_ plotted for the whole cell for 2h stress duration and up to 4h of recovery. Stress start points are marked by arrows and the recovery period is highlighted in grey. Note, stress granules dissolved after 100 min and 120 min of recovery after 1h and 2h of stress, respectively (see supplemental Movie 2). For all experiments, the data are presented as*

*mean values +/- STD and the standard deviations were calculated from the movie-wise distribution of the plotted value. The number of analyzed cells per condition (n number) is given in supplementary table S1, the experiments cells were examined in independent experiments. Source data are provided as a Source Data file.*

*
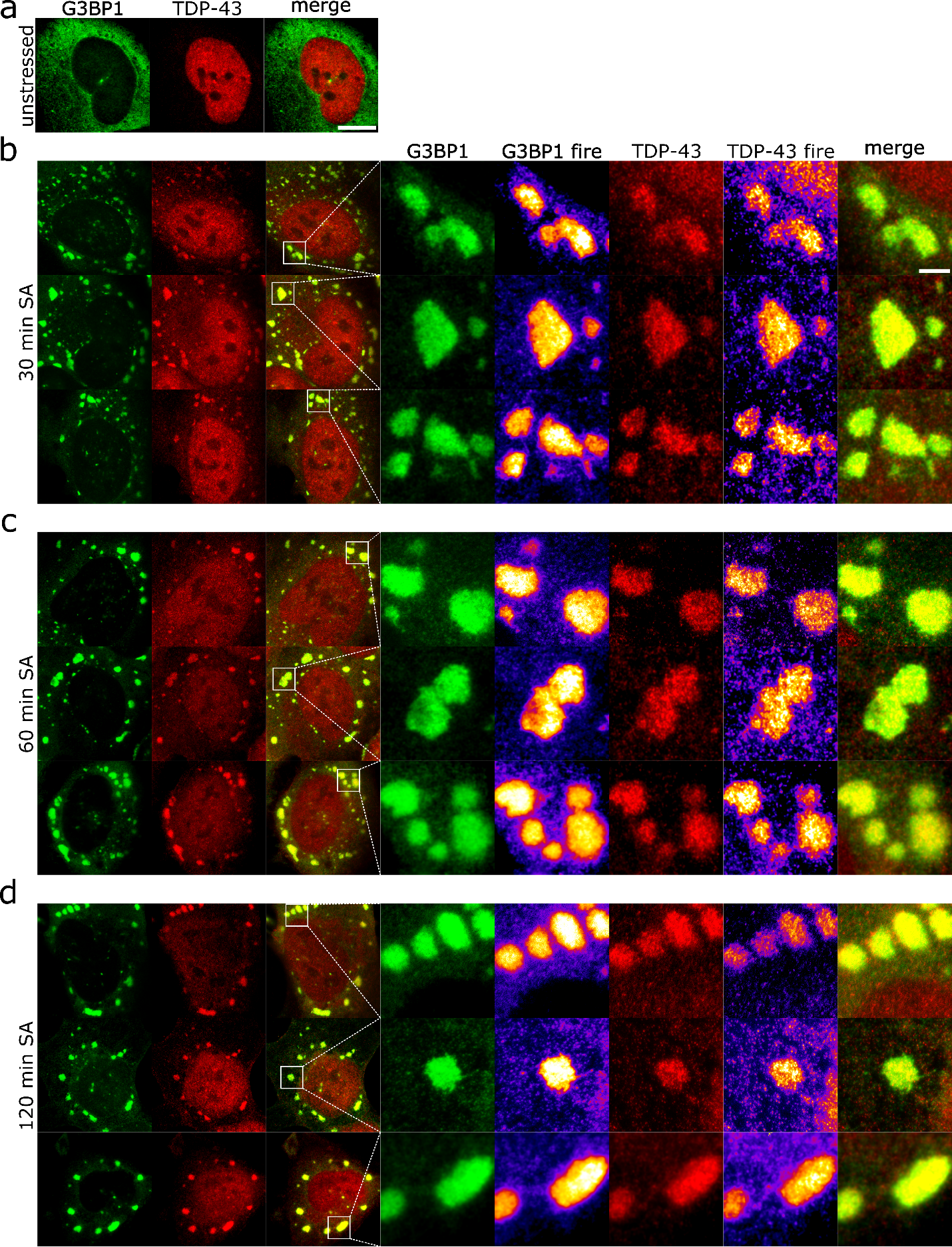
*

***Supplementary figure 16****: STED super-resolution microscopy reveals an inhomogeneous distribution of TDP-43 within stress granules.* ***a – d.*** *STED super-resolution representative images of unstressed H4 cells or imaged under different stress durations (30 min, 60 min, 120 min). Stress-granule crops are marked with a white rectangle (red: TDP-43-Atto647N, green: G3BP1-Atto594, scale bar 10 μm, 2 μm). For a better visualization of the substructure distribution a FIRE-LUT was used for the stress granule crops.*


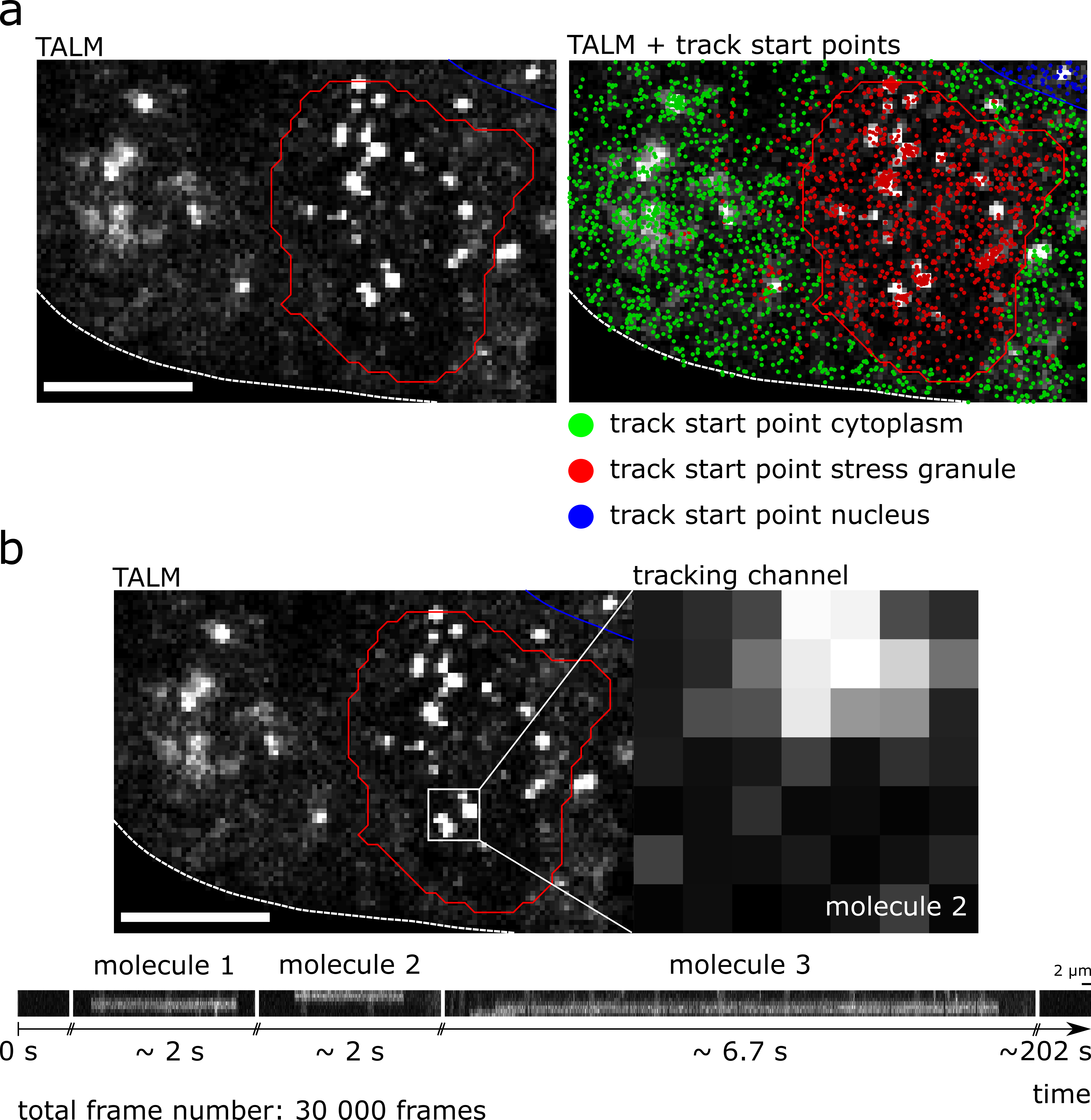


***Supplementary figure 17: a.*** *Track start point overlay with TALM images (cytoplasm: green, stress granule: red, nucleus: blue, scale bar 2 μm).* ***b.*** *Kymograph analysis of binding hotspots within stress granules shows repeated binding of TDP-43^Halo^ to the same region (scale bar 2 μm). Note that the displayed time-spans show localizations which are separated by much longer time periods without localizations, indicating single-molecule conditions.*

*
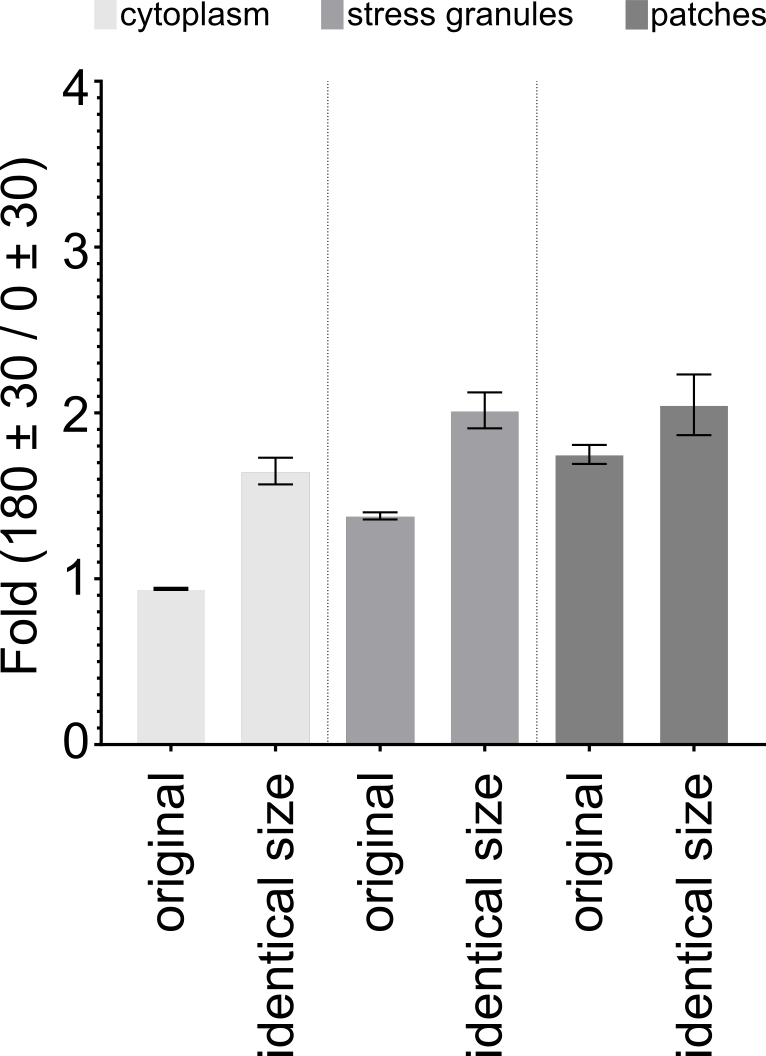
*

***Supplementary figure 18****: Quantification and comparison of the anisotropy of TDP-43 the cytoplasm, stress granules and cytoplasmic patches. Data are displayed for the highest stress condition (80-120 min) and anisotropies are compared between the regions (cytoplasm, stress granules, patches) and additionally two different methods of anisotropy determination are compared. The original data were obtained as described in the Methods part and as displayed as in figure 7 of the main paper, n = 35. For the control the anisotropy of TDP-43^Halo^ tracks was determined within a circle with a radius of 3.66 pixels. Per region, up to 3 circles were drawn and whole movies were discarded as soon as one region was too small to draw the circles for analysis, resulting in an analyzed number of n = 25 for the control data. Values are displayed as the mean +/- the STD from 50 resamplings performed with 50% of the data. Source data are provided as a Source Data file.*


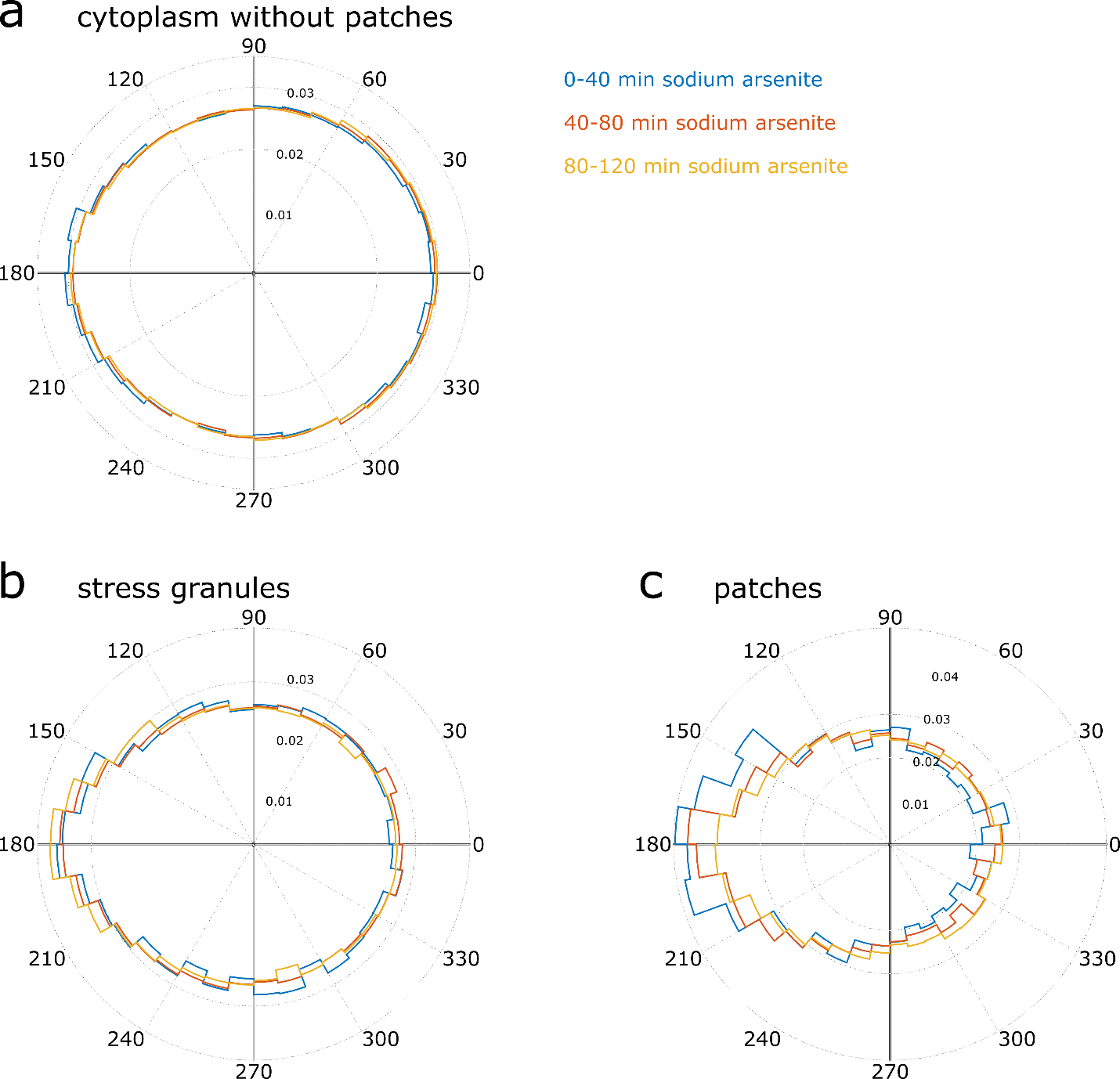


***Supplementary figure 19:*** *Angle distribution of the TDP-43^Halo^ tracks found in the cytoplasm* ***(a)****, stress granules* ***(b)*** *and cytoplasmic patches* ***(c)*** *under 0-40 min (blue), 40-80 min (orange) and 80-120 min (yellow) of sodium arsenite stress. Dashed lines indicate the respective probabilities per angular bin (width 10°).*

*.*


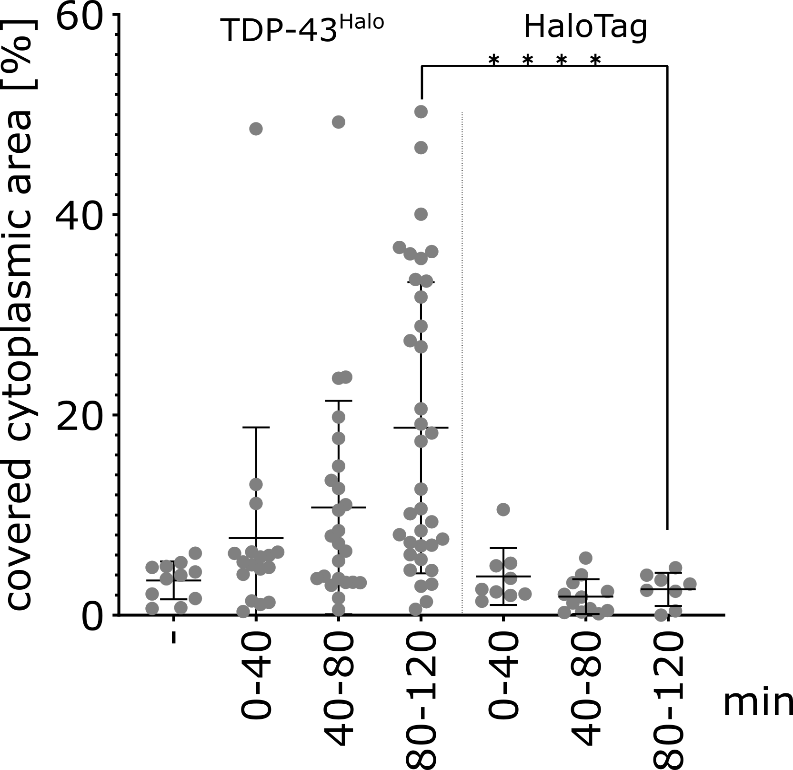


***Supplementary figure 20:*** *Relative area covered by cytoplasmic patches. The relative area for the HaloTag alone does not increase with stress duration and is similar to the value obtained for TDP-43^Halo^ in the absence of stress. Note, TDP-43^Halo^ data is the same shown in Fig. 7a and is displayed here again for a direct comparison. Statistical test: two-sided Mann-Whitney. The number of analyzed cells per condition (n number) is given in supplementary table S1, the experiments cells were examined in independent experiments. For all experiments, the data are presented as mean values +/- STD and the standard deviations were calculated from the movie-wise distribution of the plotted value, p-value = > 0.0001. Source data are provided as a Source Data file.*


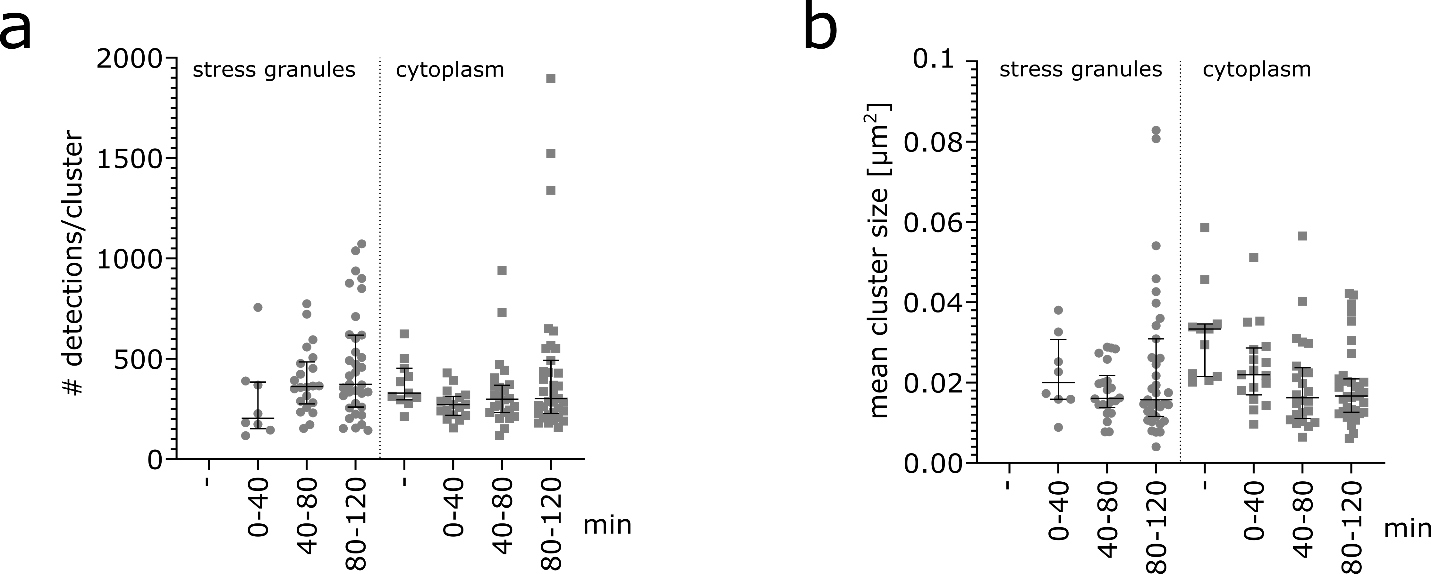


***Supplementary figure 21:*** *Analysis of TDP-43^Halo^ localization clusters found in the TALM analysis in stress granules and in the cytoplasm.* ***a.*** *Analysis of the number of detections found per cluster in stress granules and the cytoplasm under unstressed and different stress conditions. We observe similar localization within clusters in both stress granules and the cytoplasm, and the number of localizations per cluster does not change with time.* ***b.*** *Analysis of the mean cluster size [μm^2^] of clusters found in stress granules and the cytoplasm under unstressed and different stress conditions. We find a constant mean cluster size with increasing stress duration and similar mean cluster sizes in stress granules and in the cytoplasm. The number of analyzed cells per condition (n number) is given in supplementary table S1, the experiments cells were examined in independent experiments. For all experiments, the data are presented as mean values +/- STD and the standard deviations were calculated from the movie-wise distribution of the plotted value. Source data are provided as a Source Data file.*

*
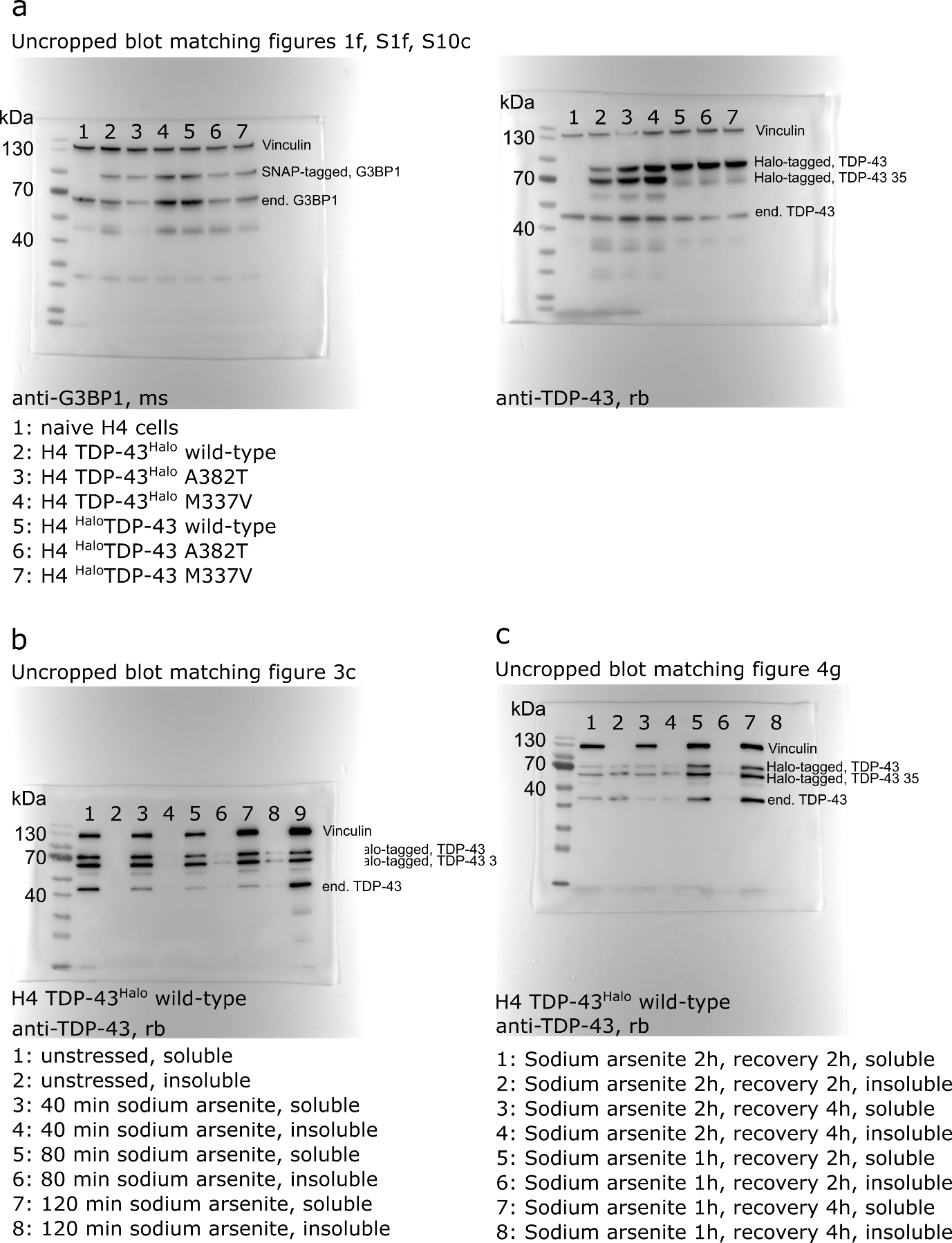
*

***Supplementary figure 22:*** *Uncropped and unprocessed western blots of blots shown in figures 1f, 3c and 4g and supplementary figures 1f and 10c.*

*Supplementary table 1: Analyzed cell numbers per tracking and experimental condition*

| **Stress time-course, sodium arsenite** | | | | |  | | |  | |  | |  | |  | |  | |  | |  |  |  |  |  |
| --- | --- | --- | --- | --- | --- | --- | --- | --- | --- | --- | --- | --- | --- | --- | --- | --- | --- | --- | --- | --- | --- | --- | --- | --- |
|  | | | | | **unstressed** | | | **20 min** | | **40 min** | | **60 min** | | **80 min** | | **100 min** | | **120 min** | |  |  |  |  |  |
| **TDP-43^Halo^ wild-type, whole cell** | | | | | 53 | | | 25 | | 22 | | 31 | | 32 | | 31 | | 32 | |  |  |  |  |  |
| **TDP-43^Halo^ wild-type, cytoplasm** | | | | | 47 | | | 24 | | 22 | | 31 | | 30 | | 30 | | 31 | |  |  |  |  |  |
| **TDP-43^Halo^ wild-type, nucleus** | | | | | 48 | | | 23 | | 20 | | 31 | | 29 | | 27 | | 24 | |  |  |  |  |  |
| **TDP-43^Halo^ wild-type, stress granule** | | | | |  | | |  | | 18 | | 26 | | 26 | | 23 | | 29 | |  |  |  |  |  |
|  | | | | |  | | |  | |  | |  | |  | |  | |  | |  |  |  |  |  |
| **TDP-43^Halo^ M337V, cytoplasm** | | | | | 46 | | | 15 | | 18 | | 22 | | 23 | | 23 | | 22 | |  |  |  |  |  |
| **TDP-43^Halo^ M337V, nucleus** | | | | | 38 | | | 14 | | 19 | | 20 | | 22 | | 22 | | 20 | |  |  |  |  |  |
| **TDP-43^Halo^ M337V, stress granule** | | | | |  | | |  | | 15 | | 20 | | 22 | | 21 | | 22 | |  |  |  |  |  |
|  | | | | |  | | |  | |  | |  | |  | |  | |  | |  |  |  |  |  |
| **TDP-43^Halo^ A382T, cytoplasm** | | | | | 52 | | | 18 | | 17 | | 22 | | 25 | | 27 | | 23 | |  |  |  |  |  |
| **TDP-43^Halo^ A382T, nucleus** | | | | | 48 | | | 19 | | 18 | | 21 | | 22 | | 24 | | 21 | |  |  |  |  |  |
| **TDP-43^Halo^ A282T, stress granule** | | | | |  | | |  | | 16 | | 22 | | 22 | | 24 | | 21 | |  |  |  |  |  |
|  | | | | |  | | |  | |  | |  | |  | |  | |  | |  |  |  |  |  |
| **^Halo^TDP-43 wild-type, whole cell** | | | | | 59 | | | 21 | | 27 | | 26 | | 27 | | 25 | | 18 | |  |  |  |  |  |
| **^Halo^TDP-43 wild-type, cytoplasm** | | | | | 52 | | | 20 | | 25 | | 24 | | 24 | | 22 | | 16 | |  |  |  |  |  |
| **^Halo^TDP-43 wild-type, nucleus** | | | | | 55 | | | 17 | | 23 | | 25 | | 26 | | 24 | | 18 | |  |  |  |  |  |
| **^Halo^TDP-43 wild-type, stress granule** | | | | |  | | |  | | 18 | | 19 | | 19 | | 19 | | 15 | |  |  |  |  |  |
|  | | | | |  | | |  | |  | |  | |  | |  | |  | |  |  |  |  |  |
| **^Halo^TDP-43 M337V, cytoplasm** | | | | | 48 | | | 12 | | 18 | | 21 | | 20 | | 18 | | 9 | |  |  |  |  |  |
| **^Halo^TDP-43 M337V, nucleus** | | | | | 45 | | | 12 | | 15 | | 21 | | 19 | | 19 | | 10 | |  |  |  |  |  |
| **^Halo^TDP-43 M337V, stress granule** | | | | |  | | |  | | 13 | | 13 | | 10 | | 12 | | 5 | |  |  |  |  |  |
|  | | | | |  | | |  | |  | |  | |  | |  | |  | |  |  |  |  |  |
| **^Halo^TDP-43 A382T, cytoplasm** | | | | | 60 | | | 15 | | 22 | | 21 | | 22 | | 19 | | 16 | |  |  |  |  |  |
| **^Halo^TDP-43 A382T, nucleus** | | | | | 63 | | | 19 | | 24 | | 23 | | 22 | | 19 | | 14 | |  |  |  |  |  |
| **^Halo^TDP-43 A282T, stress granule** | | | | |  | | |  | | 18 | | 17 | | 15 | | 11 | | 10 | |  |  |  |  |  |
|  | | | | |  | | |  | |  | |  | |  | |  | |  | |  |  |  |  |  |
| **Stress and Recovery time-course, 1h stress** | | | | | **20 min** | | | **40 min** | | **60 min** | | **80 min** | | **100 min** | | **120 min** | | **140 min** | | **160 min** | **180 min** | **200 min** | **220 min** | **240 min** |
| **TDP-43^Halo^ wild-type, whole cell** | | | | | 5 | | | 11 | | 10 | | 11 | | 16 | | 12 | | 10 | | 19 | 17 | 18 | 15 | 8 |
| **TDP-43^Halo^ wild-type, cytoplasm** | | | | | 5 | | | 11 | | 10 | | 10 | | 13 | | 12 | | 9 | | 18 | 16 | 18 | 15 | 7 |
| **TDP-43^Halo^ wild-type, nucleus** | | | | | 5 | | | 11 | | 10 | | 11 | | 14 | | 12 | | 9 | | 18 | 17 | 16 | 17 | 7 |
| **TDP-43^Halo^ wild-type, stress granule** | | | | | 3 | | | 10 | | 8 | | 8 | | 10 | |  | |  | |  |  |  |  |  |
|  | | | | |  | | |  | |  | |  | |  | |  | |  | |  |  |  |  |  |
| **Stress and Recovery time-course, 2h stress** | | | | | **20 min** | | | **40 min** | | **60 min** | | **80 min** | | **100 min** | | **120 min** | | **140 min** | | **160 min** | **180 min** | **200 min** | **220 min** | **240 min** |
| **TDP-43^Halo^ wild-type, whole cell** | | | | | 5 | | | 15 | | 11 | | 14 | | 12 | | 7 | | 15 | | 20 | 18 | 20 | 17 | 18 |
| **TDP-43^Halo^ wild-type, cytoplasm** | | | | | 7 | | | 15 | | 10 | | 14 | | 11 | | 7 | | 12 | | 19 | 18 | 18 | 15 | 15 |
| **TDP-43^Halo^ wild-type, nucleus** | | | | | 5 | | | 13 | | 9 | | 13 | | 10 | | 6 | | 11 | | 18 | 18 | 18 | 14 | 15 |
| **TDP-43^Halo^ wild-type, stress granule** | | | | | 7 | | | 9 | | 5 | | 11 | | 10 | | 6 | |  | |  |  |  |  |  |
|  |  |  |  |  | |  |  | |  | |  | |  | |  | |  | |  |  |  |  |  |  |
| **Control measurements** | | | | |  | | |  | |  | |  | |  | |  | |  | |  |  |  |  |  |
|  | | | | | **unstressed** | | | **20 min** | | **40 min** | | **60 min** | | **80 min** | | **100 min** | | **120 min** | |  |  |  |  |  |
| **TDP-43^Halo^ wild-type, whole cell, setup control** | | | | |  | | | 7 | | 12 | | 9 | | 12 | | 11 | | 12 | |  |  |  |  |  |
| **^Halo^TDP-43 wild-type, whole cell, setup control** | | | | |  | | | 7 | | 9 | | 13 | | 13 | | 11 | | 7 | |  |  |  |  |  |
| **Halo only, sodium arsenite stress, 120 min** | | | | |  | | | 15 | | 19 | | 15 | | 19 | | 20 | | 13 | |  |  |  |  |  |
| **Halo only, unstressed** | | | | | 23 | | |  | |  | |  | |  | |  | |  | |  |  |  |  |  |
| **Halo only, sorbitol stress** | | | | |  | | | 8 | | 11 | | 9 | | 11 | | 9 | | 8 | |  |  |  |  |  |
|  | | | | |  | | |  | |  | |  | |  | |  | |  | |  |  |  |  |  |
| **Stress time-course, sorbitol** | | | | |  | | |  | |  | |  | |  | |  | |  | |  |  |  |  |  |
|  | | | | | **20 min** | | | **40 min** | | **60 min** | | **80 min** | | **100 min** | | **120 min** | |  | |  |  |  |  |  |
| **TDP-43^Halo^ wild-type, whole cell** | | | | | 14 | | | 20 | | 14 | | 19 | | 14 | | 12 | |  | |  |  |  |  |  |
| **TDP-43^Halo^ wild-type, cytoplasm** | | | | | 14 | | | 20 | | 13 | | 16 | | 13 | | 10 | |  | |  |  |  |  |  |
| **TDP-43^Halo^ wild-type, nucleus** | | | | | 13 | | | 19 | | 10 | | 15 | | 14 | | 10 | |  | |  |  |  |  |  |
| **TDP-43^Halo^ wild-type, stress granule** | | | | |  | | | 11 | | 10 | | 12 | | 7 | | 7 | |  | |  |  |  |  |  |
|  | | | | |  | | |  | |  | |  | |  | |  | |  | |  |  |  |  |  |
| **Cytoplasmic patches and anisotropy analysis** | | | | |  | | |  | |  | |  | |  | |  | |  | |  |  |  |  |  |
| **Stress time-course, sodium arsenite** | | | | |  | | |  | |  | |  | |  | |  | |  | |  |  |  |  |  |
|  | | | | | **unstressed** | | | **20 - 40 min** | | **40 - 60 min** | | **60 - 80 min** | |  | |  | |  | |  |  |  |  |  |
| **TDP-43^Halo^ wild-type** | | | | | 11 | | | 17 | | 24 | | 35 | |  | |  | |  | |  |  |  |  |  |
| **HaloTag only** | | | | |  | | | 9 | | 12 | | 8 | |  | |  | |  | |  |  |  |  |  |
|  | | | | |  | | |  | |  | |  | |  | |  | |  | |  |  |  |  |  |
| **TALM localization sites analysis** | | | | |  | | |  | |  | |  | |  | |  | |  | |  |  |  |  |  |
| **Stress time-course, sodium arsenite** | | | | |  | | |  | |  | |  | |  | |  | |  | |  |  |  |  |  |
|  | | | | | **unstressed** | | | **20 - 40 min** | | **40 - 60 min** | | **60 - 80 min** | |  | |  | |  | |  |  |  |  |  |
| **TDP-43^Halo^ wild-type, cytoplasm** | | | | |  | | | 8 | | 22 | | 35 | |  | |  | |  | |  |  |  |  |  |
| **TDP-43^Halo^ wild-type, stress granules** | | | | | 11 | | | 17 | | 24 | | 35 | |  | |  | |  | |  |  |  |  |  |
| **HaloTag only, cytoplasm** | | | | |  | | | 8 | | 11 | | 8 | |  | |  | |  | |  |  |  |  |  |
| **HaloTag only, stress granules** | | | | |  | | | 4 | | 10 | | 7 | |  | |  | |  | |  |  |  |  |  |
|  | | | | |  | | |  | |  | |  | |  | |  | |  | |  |  |  |  |  |
| **Stress granule shuttling** | | | | |  | | |  | |  | |  | |  | |  | |  | |  |  |  |  |  |
| **Stress time-course, sodium arsenite** | | | | |  | | |  | |  | |  | |  | |  | |  | |  |  |  |  |  |
|  | | | | | **unstressed** | | | **20 min** | | **40 min** | | **60 min** | | **80 min** | | **100 min** | | **120 min** | |  |  |  |  |  |
| **TDP-43^Halo^ wild-type** | | | | |  | | |  | | 24 | | 33 | | 33 | | 31 | | 32 | |  |  |  |  |  |

*Supplementary table 2: Number of tracks per tracking and experimental condition*

| **Stress time-course, sodium arsenite** |  |  |  |  |  |  |  |  |  |  |  |  |
| --- | --- | --- | --- | --- | --- | --- | --- | --- | --- | --- | --- | --- |
|  | **unstressed** | **20 min** | **40 min** | **60 min** | **80 min** | **100 min** | **120 min** |  |  |  |  |  |
| **TDP-43^Halo^ wild-type, whole cell** | 149234 | 70282 | 51169 | 64490 | 58668 | 56153 | 52743 |  |  |  |  |  |
| **TDP-43^Halo^ wild-type, cytoplasm** | 94997 | 47484 | 33718 | 36832 | 34047 | 28486 | 28684 |  |  |  |  |  |
| **TDP-43^Halo^ wild-type, nucleus** | 54237 | 22798 | 13310 | 22499 | 18816 | 22176 | 19446 |  |  |  |  |  |
| **TDP-43^Halo^ wild-type, stress granule** |  |  | 4141 | 5159 | 5805 | 5491 | 4613 |  |  |  |  |  |
|  |  |  |  |  |  |  |  |  |  |  |  |  |
| **TDP-43^Halo^ M337V, cytoplasm** | 81485 | 24403 | 29698 | 33388 | 34138 | 25498 | 28663 |  |  |  |  |  |
| **TDP-43^Halo^ M337V, nucleus** | 38837 | 16730 | 19893 | 16388 | 17271 | 20074 | 18597 |  |  |  |  |  |
| **TDP-43^Halo^ M337V, stress granule** |  |  | 3290 | 3803 | 5332 | 4574 | 5170 |  |  |  |  |  |
|  |  |  |  |  |  |  |  |  |  |  |  |  |
| **TDP-43^Halo^ A382T, cytoplasm** | 95681 | 23278 | 25745 | 30417 | 33477 | 33213 | 21522 |  |  |  |  |  |
| **TDP-43^Halo^ A382T, nucleus** | 45292 | 21369 | 13042 | 16163 | 17010 | 18508 | 16939 |  |  |  |  |  |
| **TDP-43^Halo^ A282T, stress granule** |  |  | 2570 | 3860 | 5099 | 4661 | 3485 |  |  |  |  |  |
|  |  |  |  |  |  |  |  |  |  |  |  |  |
| **^Halo^TDP-43 wild-type, whole cell** | 92746 | 48861 | 62170 | 54502 | 53568 | 44256 | 30669 |  |  |  |  |  |
| **^Halo^TDP-43 wild-type, cytoplasm** | 27915 | 18766 | 27557 | 21050 | 22430 | 20978 | 13596 |  |  |  |  |  |
| **^Halo^TDP-43 wild-type, nucleus** | 64831 | 29924 | 31328 | 30415 | 27952 | 20652 | 15555 |  |  |  |  |  |
| **^Halo^TDP-43 wild-type, stress granule** |  |  | 3285 | 3037 | 3186 | 2626 | 1518 |  |  |  |  |  |
|  |  |  |  |  |  |  |  |  |  |  |  |  |
| **^Halo^TDP-43 M337V, cytoplasm** | 30052 | 4138 | 7442 | 6830 | 6865 | 5196 | 3491 |  |  |  |  |  |
| **^Halo^TDP-43 M337V, nucleus** | 45790 | 9578 | 8759 | 8904 | 7168 | 7737 | 2617 |  |  |  |  |  |
| **^Halo^TDP-43 M337V, stress granule** |  |  | 734 | 674 | 727 | 645 | 432 |  |  |  |  |  |
|  |  |  |  |  |  |  |  |  |  |  |  |  |
| **^Halo^TDP-43 A382T, cytoplasm** | 37667 | 9681 | 12055 | 9771 | 7333 | 5613 | 5488 |  |  |  |  |  |
| **^Halo^TDP-43 A382T, nucleus** | 66520 | 17091 | 12199 | 15360 | 9103 | 8026 | 7442 |  |  |  |  |  |
| **^Halo^TDP-43 A282T, stress granule** |  |  | 1431 | 1240 | 948 | 786 | 790 |  |  |  |  |  |
|  |  |  |  |  |  |  |  |  |  |  |  |  |
| **Stress and Recovery time-course, 1h stress** | **20 min** | **40 min** | **60 min** | **80 min** | **100 min** | **120 min** | **140 min** | **160 min** | **180 min** | **200 min** | **220 min** | **240 min** |
| **TDP-43^Halo^ wild-type, whole cell** | 15070 | 42249 | 27146 | 29072 | 44502 | 35566 | 33777 | 66338 | 61187 | 77846 | 67032 | 29894 |
| **TDP-43^Halo^ wild-type, cytoplasm** | 6796 | 20646 | 13880 | 14254 | 21176 | 14589 | 14466 | 38148 | 32689 | 44792 | 36892 | 20779 |
| **TDP-43^Halo^ wild-type, nucleus** | 7259 | 17094 | 10624 | 12652 | 20566 | 20405 | 18019 | 28190 | 28400 | 33054 | 30005 | 8712 |
| **TDP-43^Halo^ wild-type, stress granule** | 1015 | 4509 | 2642 | 2166 | 2760 | 572 |  |  |  |  |  |  |
|  |  |  |  |  |  |  |  |  |  |  |  |  |
| **Stress and Recovery time-course, 2h stress** | **20 min** | **40 min** | **60 min** | **80 min** | **100 min** | **120 min** | **140 min** | **160 min** | **180 min** | **200 min** | **220 min** | **240 min** |
| **TDP-43^Halo^ wild-type, whole cell** | 12454 | 29676 | 20521 | 27883 | 23149 | 16388 | 26981 | 38579 | 32663 | 36218 | 40027 | 32168 |
| **TDP-43^Halo^ wild-type, cytoplasm** | 8171 | 15505 | 10387 | 13487 | 10275 | 9292 | 15210 | 21314 | 17295 | 20904 | 21280 | 19770 |
| **TDP-43^Halo^ wild-type, nucleus** | 3239 | 11621 | 8210 | 11991 | 9720 | 5650 | 11443 | 16034 | 15003 | 15064 | 18250 | 12268 |
| **TDP-43^Halo^ wild-type, stress granule** | 1044 | 2550 | 1924 | 2405 | 3154 | 1446 |  |  |  |  |  |  |
|  |  |  |  |  |  |  |  |  |  |  |  |  |
| **Control measurements** |  |  |  |  |  |  |  |  |  |  |  |  |
|  | **unstressed** | **20 min** | **40 min** | **60 min** | **80 min** | **100 min** | **120 min** |  |  |  |  |  |
| **TDP-43^Halo^ wild-type, whole cell, setup control** |  | 21309 | 29421 | 33883 | 32522 | 26385 | 33640 |  |  |  |  |  |
| **^Halo^TDP-43 wild-type, whole cell, setup control** |  | 12396 | 14240 | 20644 | 18676 | 13897 | 12893 |  |  |  |  |  |
| **Halo only, sodium arsenite stress, 120 min** |  | 20212 | 28975 | 34249 | 32882 | 29261 | 36356 |  |  |  |  |  |
| **Halo only, unstressed** | 24599 |  |  |  |  |  |  |  |  |  |  |  |
| **Halo only, sorbitol stress** |  | 18763 | 25440 | 36370 | 31027 | 27880 | 23956 |  |  |  |  |  |
|  |  |  |  |  |  |  |  |  |  |  |  |  |
| **Stress time-course, sorbitol** |  |  |  |  |  |  |  |  |  |  |  |  |
|  | **20 min** | **40 min** | **60 min** | **80 min** | **100 min** | **120 min** |  |  |  |  |  |  |
| **TDP-43^Halo^ wild-type, whole cell** | 27981 | 47449 | 39535 | 53678 | 44047 | 27944 |  |  |  |  |  |  |
| **TDP-43^Halo^ wild-type, cytoplasm** | 17427 | 25008 | 23705 | 32440 | 27170 | 18247 |  |  |  |  |  |  |
| **TDP-43^Halo^ wild-type, nucleus** | 10015 | 19706 | 12617 | 16873 | 13767 | 7627 |  |  |  |  |  |  |
| **TDP-43^Halo^ wild-type, stress granule** |  | 2735 | 3213 | 4365 | 3110 | 2070 |  |  |  |  |  |  |
|  |  |  |  |  |  |  |  |  |  |  |  |  |
| **Cytoplasmic patches and anisotropy analysis** |  |  |  |  |  |  |  |  |  |  |  |  |
| **Stress time-course, sodium arsenite** |  |  |  |  |  |  |  |  |  |  |  |  |
|  | **unstressed** | **20 - 40 min** | **40 - 60 min** | **60 - 80 min** |  |  |  |  |  |  |  |  |
| **TDP-43^Halo^ wild-type** | 176446 | 199799 | 307135 | 399900 |  |  |  |  |  |  |  |  |
| **HaloTag only** |  | 3940 | 12310 | 6517 |  |  |  |  |  |  |  |  |
| **References** |  |  |  |  |  |  |  |  |  |  |  |  |

1. Hansen, A. S., Amitai, A., Cattoglio, C., Tjian, R. & Darzacq, X. Guided nuclear exploration increases CTCF target search efficiency. *Nat. Chem. Biol.* **16**, 257–266 (2020).

2. Hansen, A. S. *et al.* Robust model-based analysis of single-particle tracking experiments with spot-on. *Elife* **7**, 1–33 (2018).
